# Water Cooperativity Impacts Aromatic Interactions in the Aggregation of Benzene with Water

**DOI:** 10.1021/jacs.4c17315

**Published:** 2025-04-11

**Authors:** Amanda L. Steber, Farha S. Hussain, Alberto Lesarri, Timothy S. Zwier, Brooks H. Pate, Luca Evangelisti, Cristóbal Pérez

**Affiliations:** † Departamento de Química Física y Química Inorgánica, Facultad de Ciencias-I.U. CINQUIMA, 16782 Universidad de Valladolid, Valladolid E-47011, Spain; ‡ Gas Phase Chemical Physics, Sandia National Laboratories, Livermore, California 94550, United States; § Department of Chemistry, 2358 University of Virginia, Charlottesville, Virginia 22904-4319, United States; ∥ Dipartimento di Chimica “G. Ciamician”, Universita di Bologna, Bologna 40126, Italy

## Abstract

The interactions between water and aromatic rings are pervasive across various scientific and technological disciplines, including biochemistry, materials science, and environmental chemistry. In this study, we combine broadband rotational spectroscopy and quantum-chemical calculations to reveal detailed structural and binding motifs in the aggregation of benzene, as the prototypical aromatic molecule, in the presence of a few water molecules. The benzene dimer and trimer structures with up to two water molecules are conclusively identified through isotopic substitution. We observe that the π-stacking interactions are substituted by more favorable CH···π contacts, allowing the insertion of water molecules acting as bridges between aromatic rings. This induces a shortening of the O···O distances for the complexes with two water molecules compared to that of the isolated water dimer. A many-body decomposition analysis of the interaction energy reveals the interactions of water with the aromatic partners through three-body contributions. While in the prototypical hydrogen-bonded pure water clusters this contribution amounts to 20–25% of the total interaction energy, we observe a significant contribution on the order of 10% in the interactions with the benzene rings. These results experimentally rationalize the binding strength of π-systems with water.

## Graphical abstract

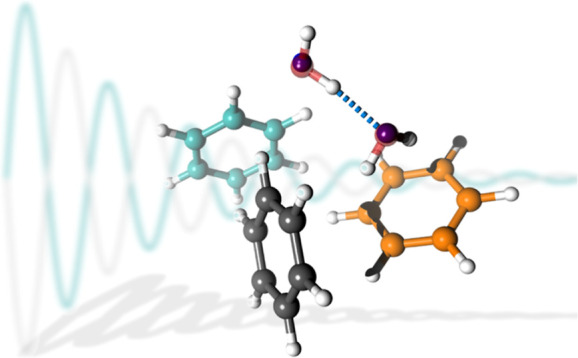

## Introduction

π-Stacking interactions, attributable to dispersion forces between aromatic rings, play a crucial role in various biological and chemical processes.^1,2^ Understanding these interactions is essential for designing functional materials,^3^ elucidating molecular recognition mechanisms,^4^ and drug research and development.^5^ Ubiquitous in biological and environmental systems, water molecules can significantly influence π-stacking interactions. The competition between π-stacking and hydrogen bonding can lead to complex, intricate structures with large implications for molecular dynamics.

Spectroscopic techniques are invaluable in probing π-stacking interactions and their interplay with water molecules. Supersonic-jet spectroscopy, in particular, offers a controlled environment free from solvent effects, allowing for an unbiased study of intrinsic molecular properties that can be directly compared with results from quantum chemistry calculations. Rotational spectroscopy, with its ability to resolve minute differences in the mass distribution of the system, has shed light on the structures and dynamics of π-stacked complexes,^6–8^ and more recently, complexes involving water molecules.^9,10^ These complexes have been subject to the distorting presence of functionalization which largely impacts the observation of the native nature of such subtle interactions.

The benzene dimer, a simple yet fundamental molecular complex, has been extensively studied as a prototypical system to understand the nature of π-stacking interactions and the strength of the resulting binding energy.^11–14^ Despite its seemingly limited binding geometries, it has been challenging to model theoretically due to the shallow potential energy surface (PES). Three binding topologies on the benzene dimer PES have been theoretically predicted corresponding to the parallel displaced π···π (PD, minimum), the CH···π (T-shaped, saddle point) and tilted T-shaped (TT, minimum), while the TT was characterized experimentally by rotational spectroscopy^13^ revealing rich internal dynamics due to the motion of the rings relative to each other. Theoretical calculations confirmed the stability of this configuration, suggesting that it is energetically competitive with the PD configuration. Beyond the benzene dimer, the benzene trimer has also been studied as a contributor to the stability of aromatic aggregation.^15,16^

Since the pioneering work by Blake and co-workers on the benzene-water dimer,^17^ the role of benzene in water aggregation, where hydrogen bonding is the driving force, has been the subject of extensive experimental investigation.^18–23^ However, the amount of experimental data on systems with π···π and CH···π interactions in the presence of water molecules is noticeably lacking.

While much previous work has concentrated on single benzene complexes with a range of water clusters up to *n* = 9, this work focuses on benzene aggregation in the presence of a controlled number of water molecules. This provides a prototypical system for studying aromatic interactions involving water. We aim to characterize benzene-rich clusters, dimer and trimer sized, when formed in the presence of a few water molecules by employing broadband rotational spectroscopy. We determine geometries and structural binding motifs of Bz_2–3_-(H_2_O)_1–2_ that illustrate the effect of water in these prototypical complexes. Furthermore, upon characterizing the experimental structures, we theoretically investigate the contributions to the binding energy of each monomer and the impact of the sequential addition of water and benzene molecules. Our results contribute to a better understanding of aromatic interactions and their interplay with water molecules.

## Results and Discussion

The rotational spectrum of benzene in water was recorded using broadband rotational spectrometers (CP-FTMW) at the University of Virginia (USA) and the University of Valladolid (Spain). Both spectrometers work similarly and are largely based on previously reported designs.^24,25^ Experimental details can be found in the methods section. As initial indicators of satisfactory cluster formation, the earlier reported Bz-(H_2_O)^23^ and pure waters clusters^26,27^ were closely monitored. To fully leverage our technique’s potential and deliver structural information, we performed several measurements with isotopically enriched water samples. In the first experiment, a sample of normal water was used (2.1 Million averages). This spectrum turned out to be very dense with 7100 transitions (signal-to-noise ratio, SNR, above 3:1) in the 2–8 GHz region as displayed in Figure 1. From the analysis of this spectrum, it was straightforward to identify intense spectral signatures attributable to a cluster of the Bz_2_-(H_2_O) size. The SNR of this spectrum was high enough (200:1) to observe the ^13^C isotopologues in natural abundance (1%). The experimental rotational parameters are reported in Table 1 for the normal species, while all the isotopic information is collected in Table S5. The statistically controlled single-substitution, in natural abundance or by using enriched samples, renders slight changes in the moments of inertia of the system that can be detected owing to the high resolution and sensitivity of current broadband spectrometers. These small changes are utilized in the Kraitchman substitution method^28^ to extract the magnitude of each positional coordinate of the substituted atom in the principal-axis system of the parent isotopic species. Moreover, this method enables an accurate structural analysis of the systems and it corroborates the proposed assignment to a specific structure based on the highly structure-sensitive isotopic shifts in the moments of inertia. A second measurement aimed at determining the position of the water molecule was performed using a 1:1 mixture H_2_^16^O/H_2_^18^O (1.5 million acquisitions). The experimentally determined atom positions are displayed as blue spheres in Figure 2 and reported in Table S6. Several initial conclusions can be drawn from the analysis of these spectra. We noticed that the spectra arising from the single ^13^C substitutions exhibited a 2-fold intensity increase relative to that expected for the natural abundance of this isotope. This is rationalized by a symmetrical geometry that makes carbon atoms equivalent in pairs. An illustration of this can be seen in the structure of Bz_2_-(H_2_O) shown in Figure 2. The observed experimental structure has a plane of symmetry formed by the oxygen atom and the C4 and C6 carbon atoms in the top ring (labeled in Figure 2) that divides the structure into two equivalent halves. Due to the large line density and the weak intensity, the rotational spectrum from ^13^C4 could not be observed in the current data set. Nevertheless, the near-total substitution structure and the excellent correlation with theoretical calculations allow for the unambiguous identification of the monohydrated benzene dimer.

**Figure 1:**
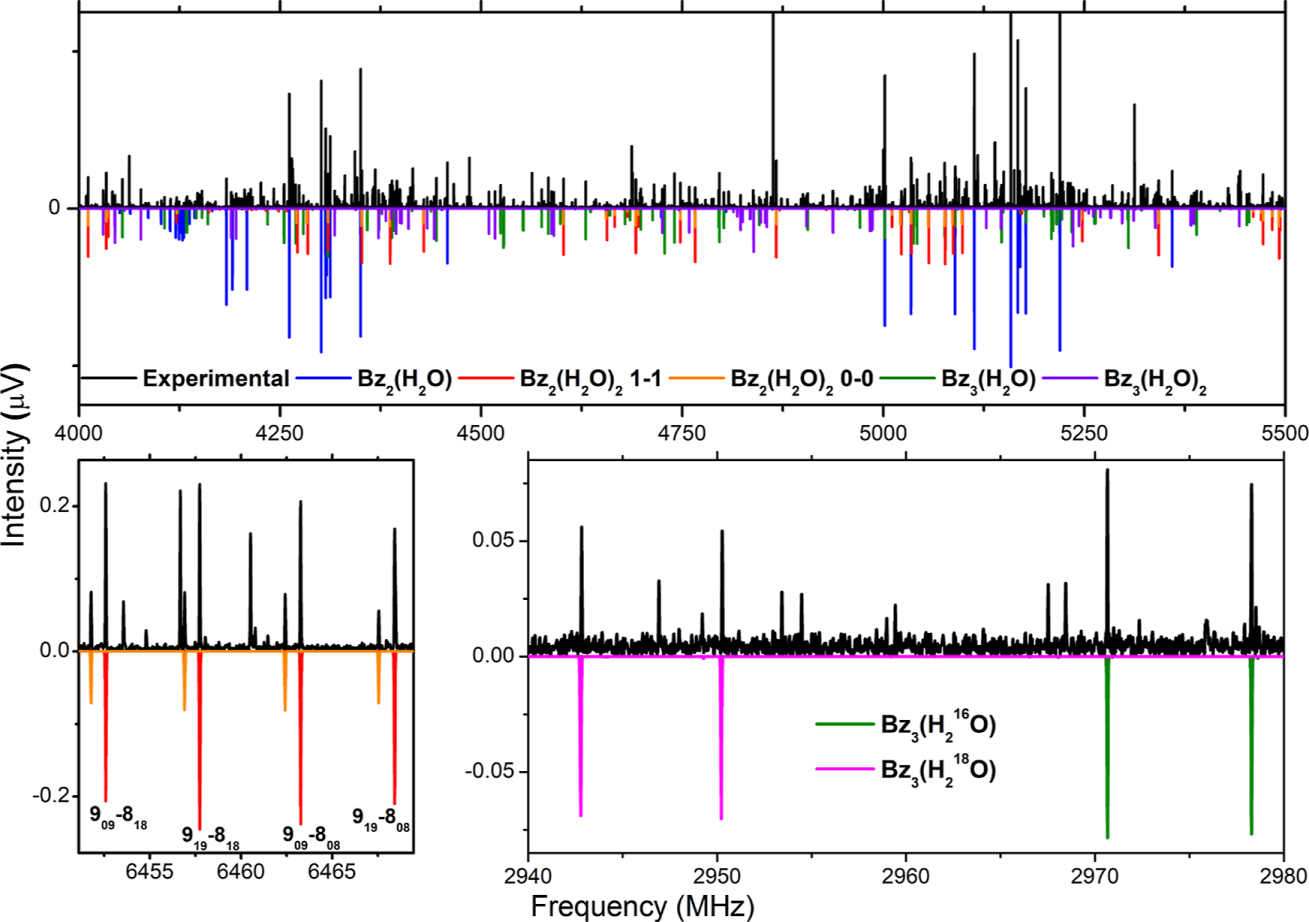
Sections of the rotational spectrum of benzene with water. Each panel features rotational transitions observed for each cluster size. The black trace is the experimental spectrum (2.1 million acquisitions). The colored traces on the negative scale are simulations (rotational temperature of 1.0 K) based on the fitted rotational parameters reported in Table 1. The rotational transitions of Bz_2_-(H_2_O)_2_ (panel bottom-left) show characteristic line splitting into two components (orange and red) that are associated with a tunneling motion arising from the interchange of two protons of a water molecule. The bottom-right panel shows the effect of isotopic substitution in the spectrum of Bz_3_-(H_2_O). The green trace is the spectrum attributed to the normal species while the magenta trace is assigned to the species exhibiting mono ^18^O substitution. See text for more details. The rotational levels involved in each transition are denoted using the standard asymmetric top notation, $J_{K_{a}K_{c}}$ , where *J* is the total angular momentum quantum number and *K*_*a*_, *K*_*c*_ are the pseudo quantum numbers for the projection of the angular momentum onto the symmetry axis (*a*- and *c*-axis) in the two limiting cases of prolate and oblate symmetric tops, respectively.

**Table 1:** Experimentally Determined Rotational Parameters for the Bz_2–3_-(H_2_O)_1–2_ Complexes^a^

	Bz_2_-(H_2_O)	Bz_2_-(H_2_O)_2_ (0)	Bz_2_-(H_2_O)_2_ (1)	Bz_3_-(H_2_O)	Bz_3_-(H_2_O)_2_
*A* (MHz)	1256.3887(21)	777.8629(14)	777.6325(14)	388.33593(13)	341.12489(19)
*B* (MHz)	439.60586(32)	427.06549(54)	427.07066(54)	291.429440(72)	235.91333(10)
*C* (MHz)	421.85227(37)	348.75480(33)	348.70674(33)	209.415020(81)	198.58388(11)
Δ_*J*_ (kHz)	0.1190(27)	0.3104(27)		0.03958(26)	0.02158(29)
Δ_*JK*_ (kHz)	0.273(37)			–0.0582(17)	
Δ_*K*_ (kHz)	8.84(30)	6.537(15)		0.1072(19)	0.0435(16)
δ_*J*_ (kHz)		–0.0902(15)		0.01114(15)	
δ_*K*_ (kHz)		2.761(33)			
σ (kHz)	10.0	9.0		4.0	9.0
*N*	67	135		242	241

a *A*, *B* and *C* are the rotational constants. Δ_*J*_, Δ_*JK*_, Δ_*K*_, δ_*J*_, δ_*K*_ are the centrifugal distortion constants in the Watson’s *A*-reduction. σ is the rms deviation of the fit, and *N* is the number of transitions in the fit.

**Figure 2:**
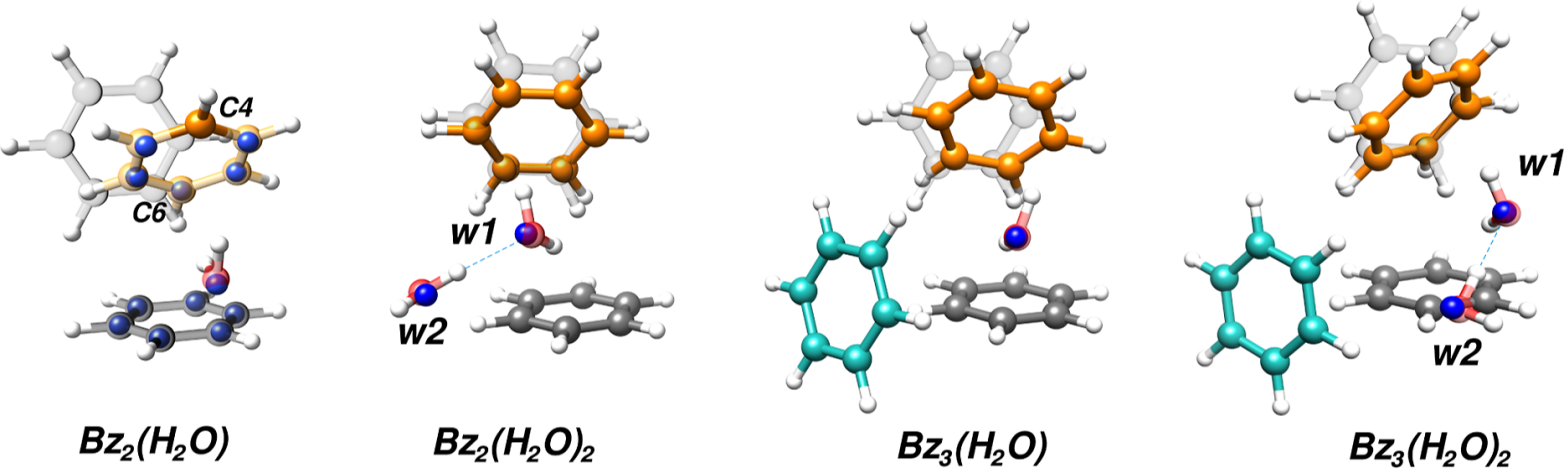
Structures of the Bz_2–3_-(H_2_O)_1–2_ clusters overlaid with the corresponding geometries from quantum-chemical calculations at the B3LYP-D3BJ/cc-pVTZ level of theory. The blue spheres represent the experimentally determined atom positions through single isotopic substitution employing the Kraitchman method. The gray (bottom) and faded rings are the structure of the tilted TT geometry of the isolated benzene dimer for comparison.

Theoretically, we performed a thorough computational search to explore the PES of the Bz_2–3_-(H_2_O)_1–2_ aggregates.

An initial screening was performed using the Conformer-Rotamer Ensemble Sampling Tool (CREST)^29^ to sample the system’s degrees of freedom. Later, the resulting pool of initial structures was further optimized using quantum-chemical calculations, particularly DFT methods. We initially chose ωB97X-D3/6-31+G­(d) as implemented in ORCA,^30,31^ which offered a reasonable trade-off between speed and accuracy. The obtained structures were subsequently reoptimized at B3LYP-D3BJ/cc-pVTZ with an energy cutoff of 2 kJ/mol. The scan for the monohydrated dimer rapidly showed satisfactory candidates for our experimental assignment. However, unlike the experimental structure, the outcome from quantum-chemical calculations exhibited a lack of symmetry. This can be rationalized through the interconversion between two equivalent minima with a low barrier. The minimal energy path (MEP) between these minima was computed using the nudged elastic band (NEB) algorithm^32^ using nine intermediate steps at the B3LYP-D3BJ/cc-pVTZ level of theory. The results show that the two minima are separated by an essentially negligible 0.05 kJ/mol barrier. The low barrier height and the excellent agreement between the experimental structure and the symmetrical transition state connecting the two equivalent minima corroborate that the zero-point energy of the ground state lies above the interconversion barrier. The interconversion barrier as well as a figure showing the two equivalent minima and the transition state structure are reported in Figure S5. Figure 2 overlays the obtained transition state with the experimentally determined structure, confirming the symmetrical structure.

Exploring the PES surface of Bz_2_-(H_2_O)_2_ turned out to be challenging. After initial optimization, most candidate structures were identified as saddle points after frequency calculations. Further optimization at B3LYP-D3BJ/cc-pVTZ using tighter convergence criteria yielded essentially two groups of structures based on their rotational constants. The three lower-energy isomers are reported in Table and Figure S2. It is worth noting that the final structures only differ in a subtle relative orientation of the benzene rings and the water dimer, which varies upon the level of calculation used. Other computational levels were also used, but given the shallow nature of the PES, a minimum obtained with one computational level often is a saddle point with another. Such challenges currently faced by theoretical methods in accurately capturing even slight changes in systems involving aromatic interactions, illustrate the crucial role of experimental data on larger molecular aggregates for benchmarking.

Experimentally, we observed a rotational spectrum with approximately equal intensity for both a- and b-type transitions. The experimental rotational parameters are reported in Table 1. There is a better agreement with the isomer Bz_2_-(H_2_O)_2_-II, despite its higher energy, both in terms of dipole moment and oxygen atom positions, which led us to assign the observed spectrum to that isomer confidently. No other isomers were found in the experimental data set. Additionally, as shown in Figure 1, each rotational transition appeared with a weaker satellite shown in orange. The 3:1 relative intensities ratios are attributed to two torsional sublevels labeled 0 and 1 arising from the interchange of two protons of a water molecule. This phenomenon usually occurs when the water molecule is free to rotate or engages only in weak hydrogen bonds. To further investigate the source of the observed splitting, we explored the proton interchange motion for each water molecule using a NEB calculation at the B3LYP-D3BJ/cc-pVTZ level of theory. The results of these trajectories are reported in Figure S6. Interestingly, both motions entail a barrier of the same order of magnitude with 12 and 16 kJ/mol for w1 and w2, respectively. Taking into account that the basic structure of the Bz_2_-(H_2_O) cluster can be identified by considering only w1, and the fact that this cluster did not show a clear hyperfine structure at the current resolution, we surmise that the observed splitting can be attributed to the internal rotation of w2 around its *C*_2_ axis, involving the rupture and formation of the hydrogen bond of the water dimer. The Kraitchman method allowed us to determine the water-oxygen atom positions in the cluster, which is overlaid with the theoretical result in Figure 2. The average value of the two levels was used for structural determinations. It is worth noting that the experimental O···O distance is 2.810(1) Å, which is remarkably shorter than 2.98(4) Å previously reported for the isolated water dimer.^33^ This is a first indication of cooperative effects with the benzene rings, which will be discussed in more detail below through multibody decomposition energy analysis.

The same overall procedure was employed to explore the PES of the Bz_3_-(H_2_O)_1–2_ aggregates. The initial screening rendered 100 structures for each cluster, subsequently optimized with a 2 kJ/mol energy cutoff. The outcome of this search is reported in Tables and Figures S3 and S4, respectively. As with the previous search, calculations yielded a set of structures with similar rotational constants and energies, resulting from slight rotations in the relative orientation of the rings and a reorientation of the water moieties. Nevertheless, the primary configuration that can be observed is that in which three benzene rings accommodate the water molecules while interacting with each other through CH···π interactions. Observing the isotopically substituted H_2_^18^O spectra enabled the determination of the oxygen position and the confirmation of the structures from the isotopic shifts as shown in Figure 1. These experimental positions show an excellent agreement with the predicted structures, especially given the difficulty in obtaining a reliable energy order and the number of observed saddle points. An overlay theory-experiment for hydrates of the benzene trimer can be seen in Figure 2. These two structures show remarkable similarities in the basic benzene-molecule network and only the water molecule w1 shifts its position to more favorably accommodate the second water moiety. An overlay highlighting this similarity is reported in Figure S8. In addition, the O···O distance in the Bz_3_-(H_2_O)_2_ is 2.891(1) Å, which gets closer to the free water dimer, indicating a weaker water–water hydrogen bond. The experimental oxygen atom positions match those of the fourth isomer in increasing order of electronic energy, namely Bz_3_-(H_2_O)_2_-IV in Table S4. This isomer is 1.80 kJ/mol higher in energy than the global minimum, but they become essentially isoenthalpic when Δ*G* is considered as the sorting criterion. Moreover, the type of spectrum observed experimentally (a and c) matches the predicted dipole moment components of isomer IV, while isomer I is predominantly b-type. Despite exhaustive efforts, we have been unable to detect isomer I. Given their structural similarity, this is likely due to the observation of vibrationally averaged structures that are well above the zero-point energy.

Once the experimental structures have been conclusively identified, it is important to gain further insight into the interactions that hold the clusters together and how this modulates the aromatic aggregation in the presence of water. A comprehensive many-body decomposition (MBD) analysis^34^ was conducted to evaluate the interaction energy of each cluster. This approach offers valuable insights into the strength of specific interactions between any pairs, triplets, etc., of monomers and their contributions to the overall interaction energy.^35,36^ To isolate each interaction of interest, all individual many-body interaction energy components between all sets of monomers were computed at the B3LYP-D3BJ/aug-cc-pVTZ. The main findings are summarized below.

The unfolding picture is that the water molecules are inserted in the structure of the benzene cluster in such a way that the π···π interactions observed for the isolated benzene dimer are substituted by a network of CH···π and/or OH···π interactions based on those CH···π observed in the tilted TT benzene dimer. Figure 2 shows the structure of the benzene dimer (faded) for comparison with the newly observed clusters. While the CH···π interaction is retained upon water addition, the tilted character of the dimer (by 60°) evolves into a symmetric structure as observed in the Bz_2_-(H_2_O) cluster. The two benzene rings are stabilized by a bifurcated OH···π interaction with the upper ring, and the oxygen atom’s lone pairs engage in a double CH···O interaction. The oxygen atom is located at the center of the ring at 3.54 Å to the four closest carbon atoms, which favors the formation of the bifurcated interaction. Moreover, the presence of water induces a 35° change from the perpendicular arrangement of the benzene rings to optimize interactions with the water monomer.

In terms of energy, one-body energy terms are not properly interactions, but they are associated with the distortion of monomers during cluster formation, resulting in relaxation or one-body energy. This energy difference reflects the disparity between monomers in their isolated gas-phase geometry and their configuration within a cluster. For conformationally rigid molecules like benzene and water, the one-body contribution is minimal. The dominant interactions are pairwise, being the strongest between the top ring (in orange) and the water moiety with 14.0 kJ/mol. The contribution to the binding energy of the CH···π interactions between rings amounts to 12.58 kJ/mol.

The addition of a second water molecule in the Bz_2_-(H_2_O)_2_ introduces changes that are worth mentioning. The basic network Bz-water-Bz is preserved in the two-water complex and the second water monomer establishes a strong, directional hydrogen bond with the first water molecule while preserving all the interactions described for the one-water complex, being the CH···π contact the most relevant between the two rings. In this case, this interaction contributes with 11.32 kJ/mol. The strongest two-body contact is the hydrogen bond between water molecules with 20.83 kJ/mol. Capturing a second water monomer allows us to study three-body interactions that are a distinctive feature of water cooperativity.^27,36^ These interactions are responsible for the three-dimensional arrangement in pure water clusters and generally amount to 20–25% of the total interaction energy in these prototypical hydrogen-bonded clusters.

Interestingly, this analysis determined the degree of water cooperativity in interactions with aromatic rings. Moreover, it allowed a comparison with purely hydrogen-bonded clusters as well as those here reported where water cooperativity is not present. We obtain 2.86 and 4.04 kJ/mol interactions for the three-body contribution in the Bz1­(gray)-w1-w2 and Bz2­(orange)-w1-w2 groups, respectively. Considering that the total binding energy for this cluster is 71.28 kJ/mol, the contribution of cooperative effects amounts to ≈10% of the overall binding energy. The results are reported in Figure 3, where the n-body contributions of the total binding energy of the cluster are illustrated. The Bz_3_-(H_2_O) complex features the basic, aforementioned unit observed for the one-water complex (rings in orange and gray overlaid with the benzene dimer in Figure 2) with the addition of the third benzene ring interacting with the bottom (gray) benzene ring through a favorable CH···π interaction. The degree of folding changes to accommodate the water molecule and the third ring while maximizing the interaction between the three rings. The MBD analysis reveals that the three rings interact with energies ranging from 7.8 to 12.6 kJ/mol, in line with smaller clusters, while the interactions with the water molecule are of similar magnitude, with the top benzene (orange) having the strongest contact with water (13.30 kJ/mol) through a bifucarted OH···π with the ring. As shown in Figure 3, the three-body contributions are negligible, confirming that the water cooperativity is not present since the cluster only contains a single water molecule. Lastly, the Bz_3_-(H_2_O)_2_ retains most of the structural and binding features already identified for the smaller predecessors. In addition, the second water monomer (w2) establishes a strong hydrogen bond with the first water (w1). Moreover, the ring in blue in Figure 2, engages w2 in a bifurcated CH···O with the lone pair of the oxygen atom. This interaction is particularly stabilizing for its kind contributing 5 kJ/mol to the total binding energy. Remarkably, the three-body interaction analysis again yielded significant contributions to the binding energy when the water dimer is involved for the three rings with 3.10, 3,5 and 1.65 kJ/mol for rings in gray, orange and blue, respectively. This unveils an important cooperative effect that contributes to the stability of the cluster, amounting to a significant ≈10% of the total interaction energy.

**Figure 3:**
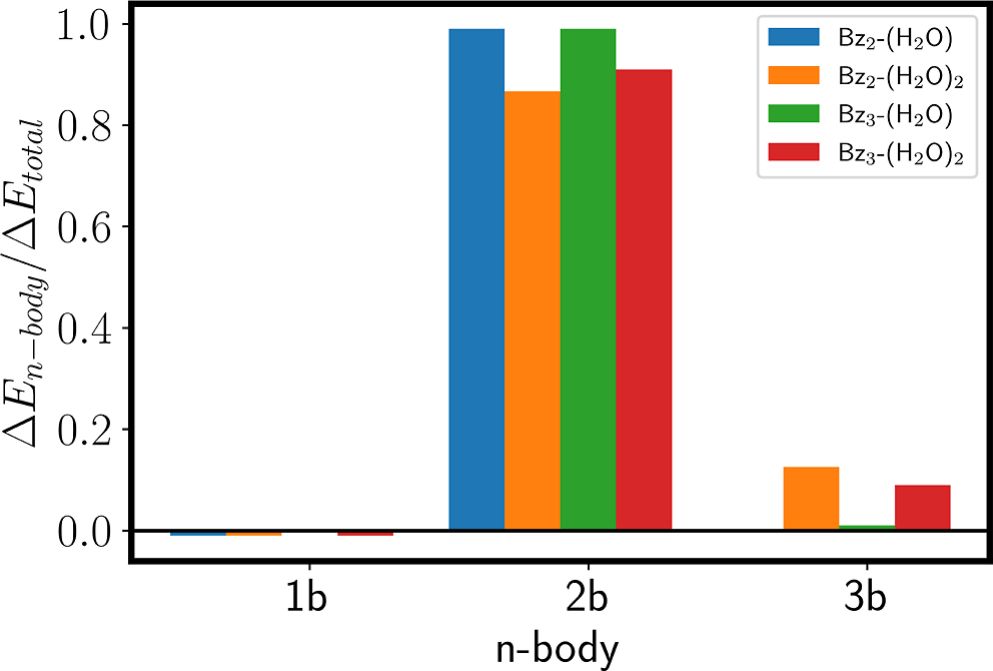
*n*-body normalized contributions to the total interaction energy for the observed Bz_2–3_-(H_2_O)_1–2_ aggregates. One-body contributions are negligible. The two- and three-body contributions are dominant and give the clusters their specific structural features. The three-body interactions are the main contributors to cooperative effects and are on the order of 10% of the total interaction energy.

## Conclusions

We have employed broadband rotational spectroscopy in tandem with quantum chemical calculations to explore the impact of water on the aggregation of benzene, the prototypical aromatic molecule. We have successfully detected and characterized benzene dimer and trimer complexes incorporating up to two water molecules. Contrary to the conventional expectation of π-stacking, where a water molecule would not be accommodated between the stacked rings, our findings reveal that the benzene dimer preferentially adopts a T-shaped configuration. This T-shaped structure, which would typically restrict water interactions to a single benzene ring, undergoes a morphological transformation into a tilted configuration. This tilted arrangement creates a favorable binding site for the water molecule to interact with both benzene rings through bifurcated CH···π and CH···O interactions. Moreover, as anticipated, the second water molecule binds to the first in a manner reminiscent of the free water dimer. However, the presence of two or three benzene molecules suppresses all but one of the tunneling pathways characteristic of the isolated water dimer, as evidenced in the Bz_2_-(H_2_O)_2_ complex. This demonstrates the considerable versatility of CH···π interactions when interacting with other molecules. It is noteworthy that the MBD analysis together with the experimentally determined shortening of the O···O distances revealed the existence of water cooperative effect (three-body interactions) with the aromatic rings and the water dimer. This happens primarily through the acceptor water in the water dimer interacting with the two benzene rings. These three-body contributions to the binding energy of the cluster are significant, being on the order of approximately 10% vs the 20% observed in pure water clusters and other hydrogen-bonded systems with polar groups. These findings confirm the role of water in molecular interactions with aromatic partners, actively stabilizing aromatic complexes beyond the well-known π-stacking mechanism.

## Experimental Section

The rotational spectra of a mixture of commercially available benzene and water were collected using the CP-FTMW spectrometers at the University of Virginia and Valladolid.^24,25^ A sample of benzene ≥ 99.0% was purchased from Merck and used without further purification. The benzene sample was diluted to 0.5% by pressurizing a tank with ca. 10 atm of pure Ne. To obtain the spectra of the clusters with water, the mixture was flown over an external reservoir of deionized water (or the desired isotopically enriched water mixture) at a total pressure of 3 bar. This benzene-water gas mixture was supersonically expanded in a vacuum chamber at ca. 10^–6^ mbar. The clusters are formed through collisional cooling at the first stages of the supersonic expansion. Subsequently, they are probed by trains of 8 chirped pulses per gas injection at 600 W of power. The molecular emission is then captured as a free-induction decay (FID), amplified, and recorded for 40 μs after each chirp excitation on a fast oscilloscope and averaged in the time domain. Finally, the frequency domain is obtained after windowing (Kaiser–Bessel) and Fourier transformation of the averaged waveform to improve the baseline resolution. We performed two measurements with H_2_^16^O (2.1 million acquisitions) and a 1:1 H_2_^16^O/H_2_^18^O mixture (1.5 million acquisitions) to favor the statistical incorporation in the structure of the clusters. The spectral analysis was performed with the programes JB95, AABS, Kra, and SPFIT. All of them are freely available.

## Supplementary Material



## Data Availability

Data available in AMSActa: https://amsacta.unibo.it/id/eprint/8054.

## Notes

Theoretical Lower-energy isomers for Bz_2–3_-(H_2_O)_1–2_. Experimental rotational parameters. Substitution structures. Internal rotation barriers for the observed isomer of Bz_2_-(H_2_O)_2_ Tables of transition frequencies. Theoretical coordinates (PDF)
