# Supplementary material for: Water Cooperativity Impacts Aromatic Interactions in the Aggregation of Benzene with Water
Source: J Am Chem Soc. 2025 Apr 11;147(23):19568–74. doi: 10.1021/jacs.4c17315 (PMC12584105; doi:10.1021/jacs.4c17315)
Supplement: Supplementary file 1 [file ja4c17315_si_001.pdf]

Supplementary Information

Water Cooperativity Impacts Aromatic Interactions in the Aggregation of Benzene with Water

Amanda L. Steber,† Farha S. Hussain,† Alberto Lesarri,† Timothy S. Zwier,‡ Brooks H. Pate,¶ Luca Evangelisti,*,§ and Cristóbal Pérez*,†

†Departamento de Química Física y Química Inorgánica, Facultad de Ciencias-I.U. CINQUIMA, Universidad de Valladolid, E-47011 Valladolid, Spain
‡Gas Phase Chemical Physics, Sandia National Laboratories, Livermore, California 94550, USA
¶Department of Chemistry, University of Virginia, Charlottesville, Virginia 22904-4319, USA
§Dipartimento di Chimica "G. Ciamician",Universita di Bologna, 40126 Bologna, Italy

E-mail: luca.evangelisti6@unibo.it; cristobal.perez@uva.es

List of Tables

Table S1: Calculated spectroscopic parameters of Bz_2- (H_2O) cluster

Table S2: Calculated spectroscopic parameters of Bz_2- $(H_2O)_2$ cluster

Table S3: Calculated spectroscopic parameters of Bz_3- (H_2O) cluster.

Table S4: Calculated spectroscopic parameters of Bz_3- $(H_2O)_2$ cluster.

Table S5: Spectroscopic parameters for Bz_2- (H_2O) cluster and its isotopologues

Table S6: Experimental atom positions for the oxygen and carbon atoms from r_s method for the Bz_2-H_2O cluster.

Table S7: Spectroscopic parameters for Bz_2-$(H_2O)_2$ cluster and its isotopologues

Table S8: Experimental atom positions for the oxygen atoms from r_s method for the Bz_2-$(H_2O)_2$

Table S9: Spectroscopic parameters for Bz_3-(H_2O) cluster and its isotopologues

Table S10: Experimental atom position for the oxygen from r_s method for the Bz_3- (H_2O)

Table S11: Spectroscopic parameters for Bz_3-$(H_2O)_2$ cluster and its isotopologues

Table S12: Experimental atom positions for the oxygen atoms from r_s method for the Bz_3- $(H_2O)_2$

Table S13: Measured rotational transitions and residuals (in MHz) of $(Bz)_2$-water Trimer.

Table S14: Measured rotational transitions and residuals (in MHz) of (^{13}C1-Benzene$)_2$-water Trimer.

Table S15: Measured rotational transitions and residuals (in MHz) of (^{13}C2-Benzene$)_2$-water Trimer.

Table S16: Measured rotational transitions and residuals (in MHz) of (^{13}C3-Benzene$)_2$-water Trimer.

Table S17: Measured rotational transitions and residuals (in MHz) of (^{13}C5-Benzene$)_2$-water Trimer

Table S18: Measured rotational transitions and residuals (in MHz) of (^{13}C6-Benzene$)_2$-water Trimer.

Table S19: Measured rotational transitions and residuals (in MHz) of (^{13}C7-Benzene$)_2$-water Trimer.

Table S20: Measured rotational transitions and residuals (in MHz) of (Benzene$)_2$-$H_2{}^{18}O$ Trimer.

Table S21: Measured rotational transitions and residuals (in MHz) of (Benzene$)_2$-(water$)_2$.

Table S22: Measured rotational transitions and residuals (in MHz) of (Benzene$)_2$-$H_2{}^{18}O$ (1)-H_2O.

Table S23: Measured rotational transitions and residuals (in MHz) of (Benzene$)_2$-H_2O-$H_2{}^{18}O$ (2).

Table S24: Measured rotational transitions and residuals (in MHz) of (Benzene$)_3$-water.

Table S25: Measured rotational transitions and residuals (in MHz) of (Benzene$)_3$-$H_2{}^{18}O$.

Table S26: Measured rotational transitions and residuals (in MHz) of (Benzene$)_3$-$(H_2O)_2$.

Table S27: Measured rotational transitions and residuals (in MHz) of (Benzene$)_3$-$H_2{}^{18}O$-H_2O.

Table S28: Measured rotational transitions and residuals (in MHz) of (Benzene$)_3$- H_2O - $H_2{}^{18}O$.

Table S29: B3LYP-D3BJ-cc-pVTZ equilibrium coordinates (Å) in the principal axis system of Bz_2-(H_2O)-I (minimum)

Table S30: B3LYP-D3BJ-cc-pVTZ equilibrium coordinates (Å) in the principal axis system of Bz_2-(H_2O)-I (Transition State)

Table S31: B3LYP-D3BJ-cc-pVTZ equilibrium coordinates (Å) in the principal axis system of Bz_2-$(H_2O)_2$-I

Table S32: B3LYP-D3BJ-cc-pVTZ equilibrium coordinates (Å) in the principal axis system of Bz_2-$(H_2O)_2$-II

Table S33: B3LYP-D3BJ-cc-pVTZ equilibrium coordinates (Å) in the principal axis system of Bz_2-$(H_2O)_2$-III

Table S34: B3LYP-D3BJ-cc-pVTZ equilibrium coordinates (Å) in the principal axis system of Bz_3-(H_2O)-I

Table S35: B3LYP-D3BJ-cc-pVTZ equilibrium coordinates (Å) in the principal axis system of Bz_3-(H_2O)-II

Table S36: B3LYP-D3BJ-cc-pVTZ equilibrium coordinates (Å) in the principal axis system of Bz_3-$(H_2O)_2$-I

Table S37: B3LYP-D3BJ-cc-pVTZ equilibrium coordinates (Å) in the principal axis system of Bz_3-$(H_2O)_2$-II

Table S38: B3LYP-D3BJ-cc-pVTZ equilibrium coordinates (Å) in the principal axis system of Bz_3-$(H_2O)_2$-III

Table S39: B3LYP-D3BJ-cc-pVTZ equilibrium coordinates (Å) in the principal axis system of Bz_3-$(H_2O)_2$-IV

List of figures

Figure S1: Lower energy isomers of Bz_2- (H_2O) cluster

Figure S2: Lower energy isomers of Bz_2-$(H_2O)_2$ cluster

Figure S3: Lower energy isomers of Bz_3- (H_2O) cluster

Figure S4: Lower energy isomers of Bz_3- $(H_2O)_2$ cluster

Figure S5: The minimal energy path (MEP) in Bz_2-(H_2O)-I cluster computed using the nudged elastic band (NEB) at the B3LYP-D3BJ-cc-pVTZ level of theory

Figure S6: The proton interchange motion in Bz_2-$(H_2O)_2$ cluster using nudged elastic band (NEB) calculation at the B3LYP-D3BJ-cc-pVTZ level of theory

Figure S7: Numbering of atom positions

Figure S8: Overlay of the observed Bz_3- (H_2O) and Bz_3- $(H_2O)_2$ complexes

Table S1: The B3LYP-D3BJ-cc-pVTZ rotational constants, principal dipole moments and relative energies of low energy isomers of Bz_2- (H_2O) cluster

	Rot. Consts (MHz)			Prin. Dipole Moment (Debye)			(kJ mol^{-1})		
Label	A	B	C	μ_a	μ_b	μ_c	ΔE_e	ZPE	ΔG
Bz_2-w-I	1294.51	455.03	439.54	-0.64	-1.75	0.13	0.0	0.0	0.33
Bz_2-w-II	1283.08	453.50	435.69	0.04	-2.02	0.27	0.6	0.60	0.0

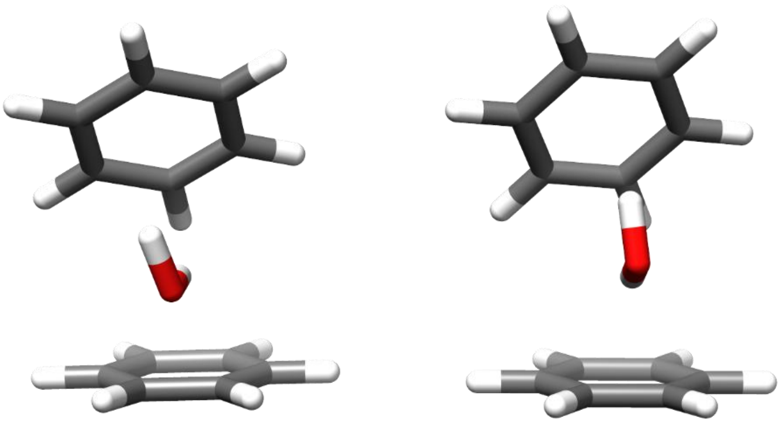

$Bz_2(H_2O)$-I ***$Bz_2(H_2O)$-II***

Figure S1: Lower energy isomers of Bz_2- (H_2O)

[Table S2](#): The B3LYP-D3BJ-cc-pVTZ rotational constants, principal dipole moments and relative energies of low energy isomers of the Bz_2-$(H_2O)_2$ cluster

	Rot. Consts (MHz)			Prin. Dipole Moment (Debye)			(kJ mol^{-1})		
Label	A	B	C	μ_a	μ_b	μ_c	ΔE_e	ZPE	ΔG
Bz_2-w_2-I	876.04	437.42	377.72	-0.43	-1.09	0.56	0.0	0.0	0.0
Bz_2-w_2-II	777.18	444.14	359.04	-0.60	-1.93	0.84	0.9	1.14	1.52
Bz_2-w_2-II	878.83	431.02	374.03	0.68	-1.30	-0.46	1.39	0.82	1.80

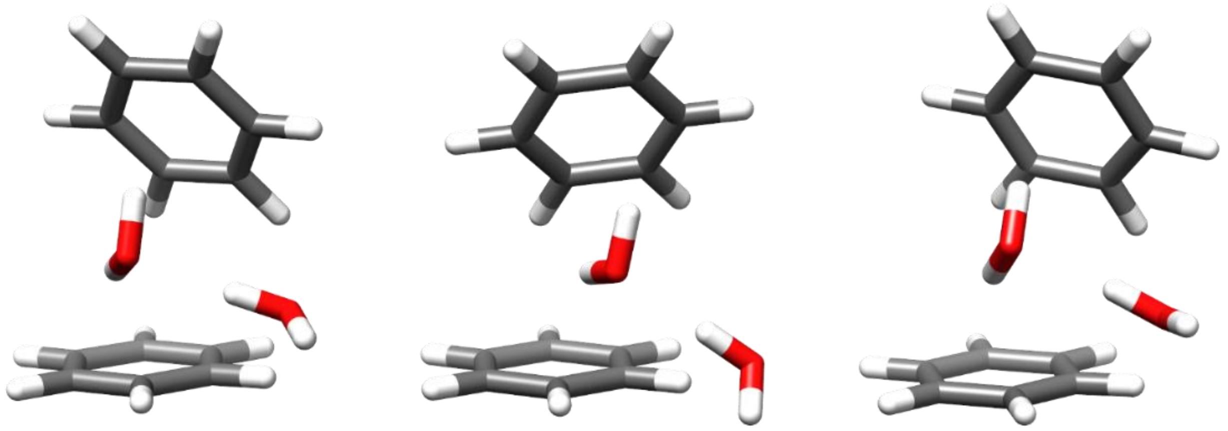

$Bz_2(H_2O)_2$-I ***$Bz_2(H_2O)_2$-II*** ***$Bz_2(H_2O)_2$-III***

[Figure S2](#): Lower energy isomers of Bz_2-$(H_2O)_2$

Table S3: The B3LYP-D3BJ-cc-pVTZ rotational constants, principal dipole moments and relative energies of low energy isomers of the Bz_3-(H_2O) cluster

	Rot. Consts (MHz)			Prin. Dipole Moment (Debye)			(kJ mol^{-1})		
Label	A	B	C	μ_a	μ_b	μ_c	ΔE_e	ZPE	ΔG
Bz_3-w-I	398.49	304.00	218.37	-1.01	0.40	-1.39	0.0	0.0	0.0
Bz_3-w-II	396.13	305.56	220.44	0.75	0.69	-1.66	0.1	0.37	0.73

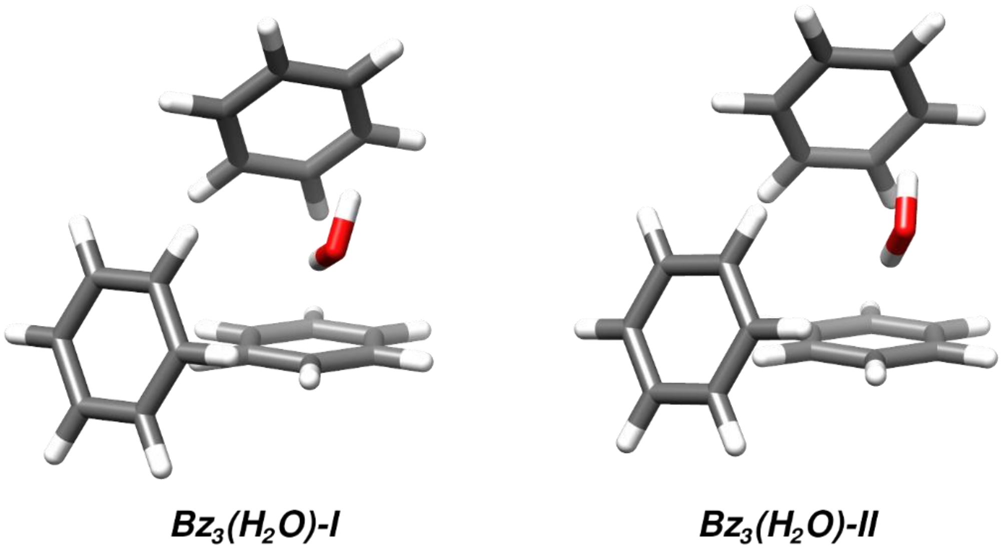

Figure S3: Lower energy isomers of Bz_3- (H_2O)

Table S4: The B3LYP-D3BJ-cc-pVTZ rotational constants, principal dipole moments and relative energies of low energy isomers of the Bz_3-$(H_2O)_2$ cluster

	Rot. Consts (MHz)			Prin. Dipole Moment (Debye)			(kJ mol^{-1})		
Label	A	B	C	μ_a	μ_b	μ_c	ΔE_e	ZPE	ΔG
Bz_3- w_2-I	355.06	253.78	208.41	-0.69	1.62	0.52	0.0	0.0	0.0
Bz_3- w_2-II	539.03	179.55	158.80	0.22	1.65	-0.65	0.48	0.80	3.50
Bz_3- w_2-III	320.44	306.35	198.55	-1.03	-1.09	-0.08	1.32	1.31	1.83
Bz_3- w_2-IV	343.61	247.46	208.67	-2.02	0.11	0.84	1.80	1.33	0.25

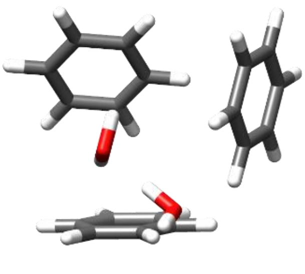

$Bz_3(H_2O)_2$-I

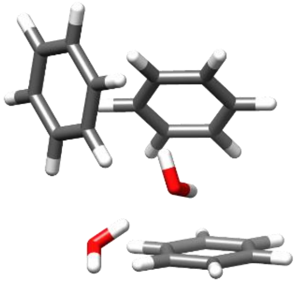

$Bz_3(H_2O)_2$-II

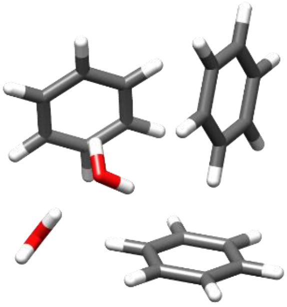

$Bz_3(H_2O)_2$-III

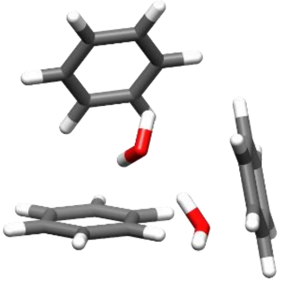

$Bz_3(H_2O)_2$-IV

Figure S4: Lower energy isomers of Bz_3- $(H_2O)_2$

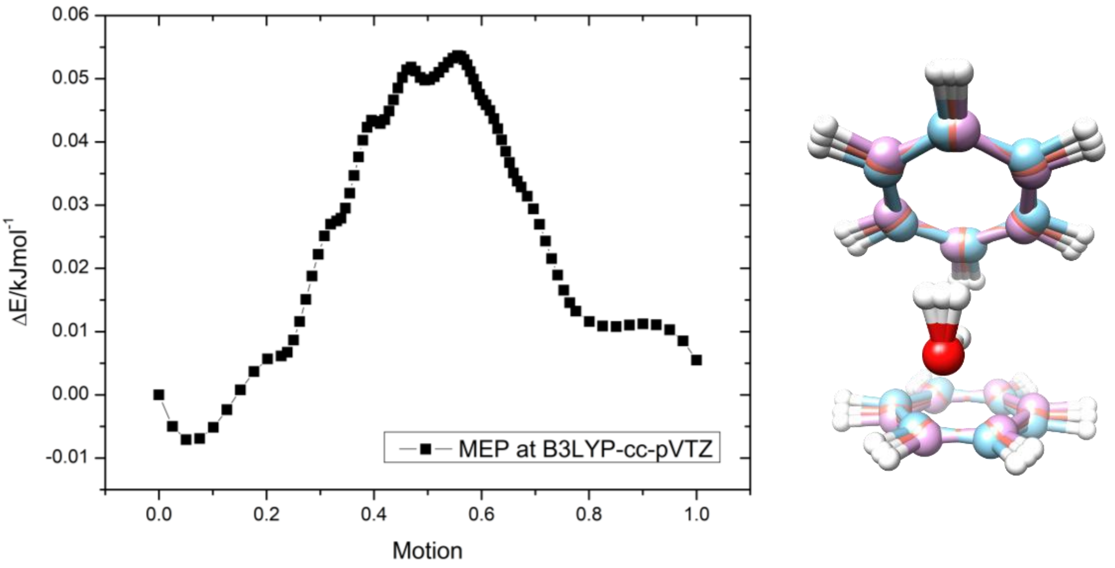

Figure S5: The minimal energy path (MEP) between two equivalent minima (blue and pink structures) of Bz_2-(H_2O)-I computed using the nudged elastic band (NEB) at the B3LYP-D3BJ-cc-pVTZ level of theory. The red structure is the obtained transition state that matches the experimental structure.

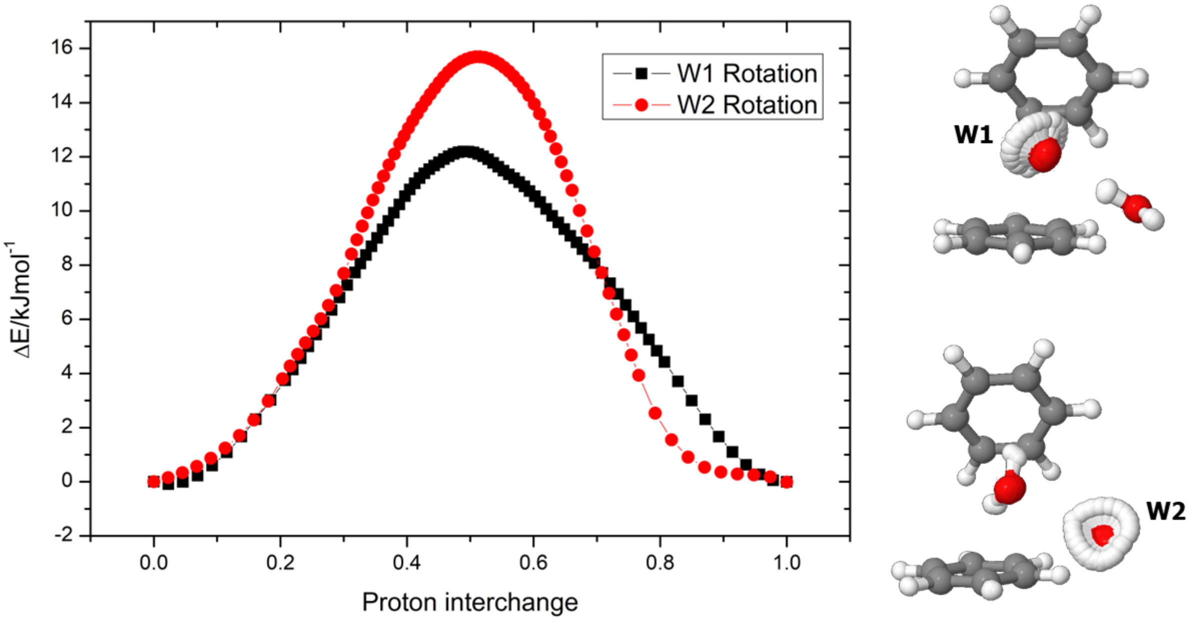

Figure S6: The proton interchange motion for each water molecule in Bz_2-$(H_2O)_2$ cluster using nudged elastic band (NEB) calculation at the B3LYP- D3BJ-cc-pVTZ level of theory.

Table S5: Spectroscopic parameters determined from fitting the measured transition frequencies for the parent Bz_2- (H_2O) and the observed isotopologues.

Parameter	Normal
A / MHz	1256.3887(21)
B / MHz	439.60586(32)
C / MHz	421.85227(37)
D_J / kHz	0.1190(27)
D_K / kHz	8.84(30)
D_{JK} / kHz	0.273(37)
σ / kHz	10
N	67

	^{13}C1	^{13}C2	^{13}C3
A / MHz	1250.4530(15)	1250.78(14)	1250.62(40)
B / MHz	436.60108(28)	437.97716(36)	438.15030(87)
C / MHz	419.75311(20)	420.08172(36)	420.78701(83)
σ / kHz	7	7	12
N	26	36	18

	^{13}C5	^{13}C6	^{13}C7
A / MHz	1251.03(32)	1253.2500(71)	1252.16(30)
B / MHz	436.15430(59)	439.32249(44)	435.92550(60)
C / MHz	418.41980(61)	421.23514(39)	418.90740(65)
σ / kHz	8	7	5
N	18	23	16

* D_J /kHz [0.119024377], D_K /kHz [8.839634048] , D_{JK} /kHz [0.273011474].

	$H_2{}^{18}O$
A / MHz	1217.25760(90)
B / MHz	438.91223(45)
C / MHz	416.71762(36)
σ / kHz	7
N	24

* D_J /kHz [0.119024377], D_K /kHz [8.839634048] , D_{JK} /kHz [0.273011474]

Table S6: Experimental atom positions for the oxygen and carbon atoms from r_s method for the Bz_2-H_2O cluster.

	|a|	|b|	|c|
^{13}C (1)	2.44883(64)	0	1.39066(11)
^{13}C (2)	1.93918(59)	1.13347(10)	0.72955(16)
^{13}C (3)	1.58060(20)	0.72318(44)	1.15881(20)
^{13}C (5)	2.93297(09)	1.10944(23)	0.71819(04)
^{13}C (6)	0.86337(07)	1.00698(06)	0
^{13}C (7)	2.89661(8)	0.19155(12)	1.15732(21)
^{18}O	0.95100(16)	2.55858(06)	0

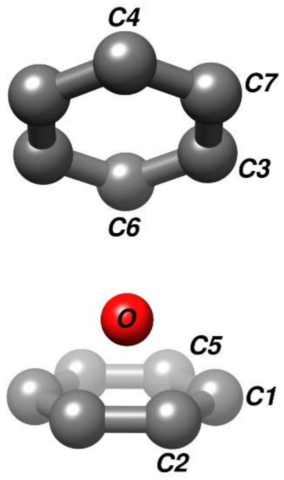

Figure S7: Numbering of atom positions

Table S7: Spectroscopic parameters determined from fitting the measured transition frequencies for the parent Bz_2-$(H_2O)_2$ oxygen isotopologues.

Parameter	normal	
	0←0	1←1
A / MHz	777.8629(14)	777.6325(14)
B / MHz	427.06549(54)	427.07066(54)
C / MHz	348.75480(33)	348.70674(33)
D_J / kHz	0.3104(27)	
D_K / kHz	6.537(15)	
d_j / kHz	-0.0902(15)	
d_k / kHz	2.761(33)	
σ / kHz	9	
N	135	

	$H_2{}^{18}O$ (1)	$H_2{}^{18}O$ (2)
A / MHz	766.9730(80)	748.332(11)
B / MHz	424.81520(49)	427.04240(52)
C / MHz	347.05053(28)	342.64390(64)
D_J / kHz		0.3602(33)
σ / kHz	11	10
N	39	34

* D_J /kHz [0.31043268], D_{JK} /kHz [6.536927004], d_j /kHz [-0.090188597], d_k /kHz [2.761196475]

Table S8: Experimental atom positions for the oxygen atoms from r_s method for the Bz_2-$(H_2O)_2$

	|a|	|b|	|c|
^{18}O (1)	1.01703(17)	1.56782(11)	1.46943(12)
^{18}O (2)	0.25595(06)	3.59396(04)	0

Table S9: Spectroscopic parameters determined from fitting the measured transition frequencies for the parent Bz_3-(H_2O)cluster and oxygen isotopologue.

Parameter	normal
A / MHz	388.33593(13)
B / MHz	291.429440(72)
C / MHz	209.415020(81)
D_J / kHz	0.03958(26)
D_K / kHz	0.1072(19)
D_{JK} / kHz	-0.0582(17)
d_j / kHz	0.01114(15)
σ / kHz	4
N	242

	(Benzene)$_3$ – water(^{18}O)
A / MHz	384.529250(54)
B / MHz	289.409840(34)
C / MHz	209.131600(34)
σ / kHz	4
N	195

* D_J /kHz [0.039578237], D_K /kHz [0.107246857], D_{JK} /kHz [-0.058157093], d_j /kHz [0.011140616

Table S10: Experimental atom position for the oxygen atom from r_s method for the Bz_3-(H_2O)

	|a|	|b|	|c|
^{18}O	0.78535(19)	1.00279(15)	2.34147(06)

Table S11: Spectroscopic parameters determined from fitting the measured transition frequencies for the parent Bz_3-$(H_2O)_2$ cluster and oxygen isotopologues.

Parameter	normal
A / MHz	341.12489(19)
B / MHz	235.91333(10)
C / MHz	198.58388(11)
D_J / kHz	0.02158(29)
D_K / kHz	0.0435(16)
σ / kHz	9
N	241

	(Benzene)$_3$ – water(^{18}O1)	(Benzene)$_3$ – water(^{18}O2)
A / MHz	338.8977(11)	335.6334(13)
B / MHz	234.133080(90)	233.29724(11)
C / MHz	198.020070(88)	198.393190(94)
σ / kHz	5	6
N	72	77

* D_J /kHz [0.02158498], D_K /kHz [0.043507314]

Table S12: Experimental atom positions for the oxygen atoms from r_s method for the Bz_3- $(H_2O)_2$

	|a|	|b|	|c|
^{18}O (1)	1.85347(09)	0.41478(04)	2.18042(08)
^{18}O (2)	0.73948(02)	0.79529(02)	3.40067(05)

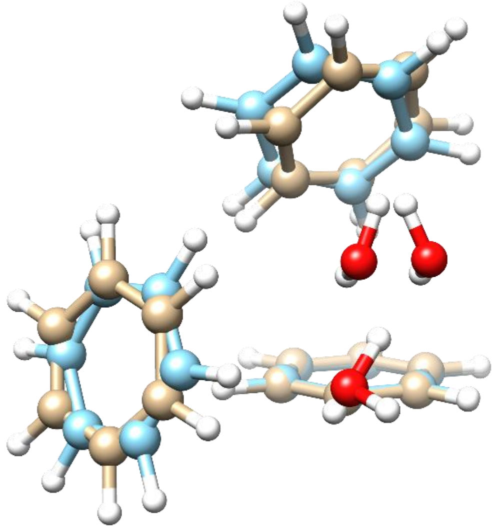

Figure S8: Overlay of the observed Bz_3-(H_2O) and Bz_3-($H_2O)_2$ complexes in blue and light maroon, respectively.

Table S13: Measured rotational transitions and residuals (in MHz) of $(Bz)_2$-water Trimer.

J’	Ka’	Kc’	←	J’’	Ka’’	Kc’’	υ_{obs}	υ_{obs}-υ_{cal}
5	1	5	←	4	1	4	4261.6254	0.003
5	0	5	←	4	0	4	4301.5272	0
5	1	4	←	4	1	3	4350.3339	0.004
4	1	4	←	3	1	3	3409.73	0.001
4	0	4	←	3	0	3	3442.9448	0.001
4	1	3	←	3	1	2	3480.7258	0
3	1	3	←	2	1	2	2557.5491	-0.002
3	0	3	←	2	0	2	2583.2179	0.001
3	1	2	←	2	1	1	2610.8056	-0.004
6	1	6	←	5	1	5	5113.1737	0.002
6	0	6	←	5	0	5	5158.7054	-0.001
6	1	5	←	5	1	4	5219.5375	0.006
7	1	7	←	6	1	6	5964.3238	0
7	0	7	←	6	0	6	6014.2491	0
7	1	6	←	6	1	5	6088.2422	0.007
8	1	8	←	7	1	7	6815.0331	0.001
8	0	8	←	7	0	7	6867.9742	0
8	1	7	←	7	1	6	6956.3403	0.004
9	1	9	←	8	1	8	7665.265	0.005
9	0	9	←	8	0	8	7719.7752	0.011
9	1	8	←	8	1	7	7823.7141	-0.002
4	2	3	←	3	2	2	3445.5986	0.027
4	3	2	←	3	3	1	3446.3561	0.009
4	3	1	←	3	3	0	3446.3561	-0.007

4	2	2	←	3	2	1	3448.4063	-0.022
5	2	4	←	4	2	3	4306.5938	0.009
5	4	2	←	4	4	1	4307.8238	0
5	4	1	←	4	4	0	4307.8238	0
5	3	3	←	4	3	2	4308.1928	0.032
5	3	2	←	4	3	1	4308.1928	-0.028
5	2	3	←	4	2	2	4312.2816	-0.005
6	2	5	←	5	2	4	5167.3465	0.001
6	2	4	←	5	2	3	5177.2799	-0.002
7	2	6	←	6	2	5	6027.8008	-0.001
7	2	5	←	6	2	4	6043.5895	-0.003
8	2	7	←	7	2	6	6887.9026	-0.004
8	2	6	←	7	2	5	6911.3495	0.002
9	2	8	←	8	2	7	7747.6034	-0.006
9	3	7	←	8	3	6	7756.8906	0.029
9	3	6	←	8	3	5	7758.1444	-0.042
9	2	7	←	8	2	6	7780.6111	0.008
3	2	2	←	2	2	1	2584.3545	0
3	2	1	←	2	2	0	2585.5018	0.002
2	2	1	←	1	1	0	4190.8714	-0.004
2	2	0	←	1	1	1	4208.9102	-0.005
6	0	6	←	5	1	5	4458.1975	0.01
5	1	5	←	4	0	4	5002.0363	-0.01
3	2	2	←	2	1	1	5034.5542	-0.011
3	2	1	←	2	1	2	5089.2521	-0.005
7	0	7	←	6	1	6	5359.2681	0.001
6	1	6	←	5	0	5	5813.6878	-0.002
4	2	3	←	3	1	2	5869.3391	0.012
4	2	2	←	3	1	3	5980.1295	-0.005
7	1	7	←	6	0	6	6619.2849	-0.021
5	2	4	←	4	1	3	6695.1997	0.014
4	1	4	←	3	0	3	4183.3557	-0.013
2	1	2	←	1	0	1	2521.9296	-0.001
3	2	2	←	3	1	3	2530.2716	-0.003
4	0	4	←	3	1	3	2669.2998	-0.003
6	1	5	←	5	2	4	2874.6536	-0.021
3	1	3	←	2	0	2	3356.863	0.005
5	0	5	←	4	1	4	3561.1188	0.016
7	1	6	←	6	2	5	3795.5564	-0.009
6	3	3	←	6	2	4	4113.3084	-0.007
5	2	3	←	4	1	4	6882.6951	0.001
8	1	8	←	7	0	7	7420.0869	-0.001
6	2	5	←	5	1	4	7512.2108	0.01

Table S14: Measured rotational transitions and residuals (in MHz) of (^{13}C1-Benzene)$_2$-water Trimer.

J’	Ka’	Kc’	←	J’’	Ka’’	Kc’’	υ_{obs}	υ_{obs}-υ_{cal}
4	0	4	←	3	0	3	3422.8009	-0.000
5	0	5	←	4	0	4	4276.5595	0.007

5	2	4	←	4	2	3	4281.1248	-0.000
5	2	3	←	4	2	2	4286.2925	0.008
6	1	6	←	5	1	5	5085.4807	0.008
6	0	6	←	5	0	5	5129.0239	-0.003
6	2	5	←	5	2	4	5136.8414	-0.002
6	1	5	←	5	1	4	5186.4192	-0.001
7	1	7	←	6	1	6	5932.1127	0.012
7	1	6	←	6	1	5	6049.7109	-0.012
8	1	7	←	7	1	6	6912.4779	-0.003
3	2	2	←	2	1	1	5010.4671	0.005
6	1	6	←	5	0	5	5788.6159	-0.016
4	2	3	←	3	1	2	5841.4860	0.005
5	1	4	←	4	1	3	4322.6588	-0.010
4	1	4	←	3	1	3	3391.1842	0.006
5	1	5	←	4	1	4	4238.4947	0.012
6	2	4	←	5	2	3	5145.8391	0.001
7	0	7	←	6	0	6	5980.0173	0.003
7	2	6	←	6	2	5	5992.2835	-0.004
7	2	5	←	6	2	4	6006.5859	-0.000
8	2	7	←	7	2	6	6847.4055	-0.004
9	1	9	←	8	1	8	7624.1099	0.007
9	0	9	←	8	0	8	7676.8828	0.000
5	2	4	←	4	1	3	6664.0472	-0.003
8	1	8	←	7	0	7	7390.0071	-0.005

Table S15**:** Measured rotational transitions and residuals (in MHz) of (^{13}C2-Benzene)$_2$-water Trimer.

J’	Ka’	Kc’	←	J’’	Ka’’	Kc’’	υ_{obs}	υ_{obs}-υ_{cal}
4	1	4	←	3	1	3	3395.841	0.004
4	0	4	←	3	0	3	3429.2886	0.001
4	2	3	←	3	2	2	3431.9781	0.009
4	3	2	←	3	3	1	3432.7715	0.01
4	3	1	←	3	3	0	3432.7715	-0.007
4	2	2	←	3	2	1	3434.8737	-0.013
4	1	3	←	3	1	2	3467.3887	-0.011
5	1	5	←	4	1	4	4244.2533	0.007
5	0	5	←	4	0	4	4284.4123	0
5	2	4	←	4	2	3	4289.5772	0.002
5	4	2	←	4	4	1	4290.8349	-0.005
5	4	1	←	4	4	0	4290.8349	-0.006
5	2	3	←	4	2	2	4295.3952	0
5	1	4	←	4	1	3	4333.6579	-0.002
6	1	6	←	5	1	5	5092.3095	0.004
6	0	6	←	5	0	5	5138.1105	0.004
6	2	4	←	5	2	3	5157.0697	0.004
6	1	5	←	5	1	4	5199.5125	0.001

7	1	7	←	6	1	6	5939.9649	0.005
7	0	7	←	6	0	6	5990.1344	0.002
7	2	6	←	6	2	5	6003.9596	-0.001
7	2	5	←	6	2	4	6020.0658	-0.009
7	1	6	←	6	1	5	6064.8432	-0.009
8	1	8	←	7	1	7	6787.1708	0.008
8	0	8	←	7	0	7	6840.3072	0.001
8	2	7	←	7	2	6	6860.6345	-0.005
8	5	4	←	7	5	3	6865.5495	0.002
8	5	3	←	7	5	2	6865.5495	0.002
8	4	5	←	7	4	4	6866.2178	0.011
8	4	4	←	7	4	3	6866.2178	0.003
8	2	6	←	7	2	5	6884.5461	-0.007
8	1	7	←	7	1	6	6929.5773	0
9	1	9	←	8	1	8	7633.8521	-0.025
9	2	7	←	8	2	6	7750.5696	0.012
9	1	8	←	8	1	7	7793.5732	0.007
3	0	3	←	2	0	2	2573.0000	0.004

Table S16: Measured rotational transitions and residuals (in MHz) of (^{13}C3-Benzene)$_2$-water Trimer.

J’	Ka’	Kc’	←	J’’	Ka’’	Kc’’	υ_{obs}	υ_{obs}-υ_{cal}
4	1	4	←	3	1	3	3400.4577	0.010
4	0	4	←	3	0	3	3432.9767	0.007
4	2	3	←	3	2	2	3435.5127	0.016
4	2	2	←	3	2	1	3438.2198	-0.025
4	1	3	←	3	1	2	3469.8937	0.009
5	1	5	←	4	1	4	4250.0467	0.006
5	0	5	←	4	0	4	4289.1412	0.000
5	2	4	←	4	2	3	4294.0082	0.003
5	2	3	←	4	2	2	4299.4827	-0.007
5	1	4	←	4	1	3	4336.7987	-0.000
6	0	6	←	5	0	5	5143.9449	-0.014
6	1	5	←	5	1	4	5203.3484	0.020
7	1	7	←	6	1	6	5948.1821	0.004
7	0	7	←	6	0	6	5997.2021	0.001
7	2	5	←	6	2	4	6025.4191	-0.017
7	1	6	←	6	1	5	6069.3804	0.002
8	1	8	←	7	1	7	6796.6353	0.009
8	0	8	←	7	0	7	6848.6682	-0.017

Table S17: Measured rotational transitions and residuals (in MHz) of (^{13}C5-Benzene)$_2$-water Trimer.

J’	Ka’	Kc’	←	J’’	Ka’’	Kc’’	υ_{obs}	υ_{obs}-υ_{cal}
4	0	4	←	3	0	3	3415.4034	-0.003
4	2	3	←	3	2	2	3418.0462	0.011
4	3	2	←	3	3	1	3418.8174	0.006
4	3	1	←	3	3	0	3418.8171	-0.010

4	2	2	←	3	2	1	3420.8778	-0.015
4	1	3	←	3	1	2	3453.1515	0.000
5	1	5	←	4	1	4	4227.2523	0.002
5	0	5	←	4	0	4	4267.1029	-0.003
5	2	4	←	4	2	3	4272.1589	-0.004
5	2	3	←	4	2	2	4277.8777	0.009
5	1	4	←	4	1	3	4315.8647	0.003
6	1	6	←	5	1	5	5071.9177	-0.006
6	0	6	←	5	0	5	5117.3883	-0.013
6	1	5	←	5	1	4	5178.1759	0.006
7	1	7	←	6	1	6	5916.2142	0.012
7	0	7	←	6	0	6	5966.0627	0.002
7	2	5	←	6	2	4	5995.4018	-0.004
7	1	6	←	6	1	5	6039.9800	0.000

Table S18: Measured rotational transitions and residuals (in MHz) of (^{13}C6-Benzene)$_2$-water Trimer.

J’	Ka’	Kc’	←	J’’	Ka’’	Kc’’	υ_{obs}	υ_{obs}-υ_{cal}
5	1	5	←	4	1	4	4256.2307	-0.004
5	0	5	←	4	0	4	4296.7899	-0.000
5	1	4	←	4	1	3	4346.6041	-0.003
6	1	6	←	5	1	5	5106.6839	0.006
6	0	6	←	5	0	5	5152.9038	0.007
6	1	5	←	5	1	4	5215.0302	0.000
7	1	7	←	6	1	6	5956.7102	0.002
7	0	7	←	6	0	6	6007.3016	-0.002
7	2	6	←	6	2	5	6021.4082	0.001
7	1	6	←	6	1	5	6082.9246	-0.008
5	1	5	←	4	0	4	4992.4460	-0.003
7	0	7	←	6	1	6	5357.8783	0.013
5	0	5	←	4	1	4	3560.5596	-0.016
4	1	4	←	3	1	3	3405.4445	0.008
4	0	4	←	3	0	3	3439.2167	-0.006
4	1	3	←	3	1	2	3477.7603	-0.006
5	2	4	←	4	2	3	4302.0566	0.001
5	2	3	←	4	2	2	4307.9801	-0.012
7	2	5	←	6	2	4	6037.8305	-0.008
8	1	8	←	7	1	7	6806.2809	0.002
8	0	8	←	7	0	7	6859.8213	-0.005
8	2	7	←	7	2	6	6880.5638	0.005
8	1	7	←	7	1	6	6950.2212	0.015

Table S19: Measured rotational transitions and residuals (in MHz) of (^{13}C7-Benzene)$_2$-water Trimer.

J’	Ka’	Kc’	←	J’’	Ka’’	Kc’’	υ_{obs}	υ_{obs}-υ_{cal}
4	1	4	←	3	1	3	3384.7401	-0.003
4	0	4	←	3	0	3	3416.6686	-0.002
5	1	5	←	4	1	4	4230.4292	-0.001

5	0	5	←	4	0	4	4268.8474	-0.009
5	2	4	←	4	2	3	4273.5232	0.014
5	2	3	←	4	2	2	4278.7548	-0.001
6	0	6	←	5	0	5	5119.7457	0
5	1	4	←	4	1	3	4315.4646	-0.003
6	2	5	←	5	2	4	5127.6927	-0.003
6	2	4	←	5	2	3	5136.8484	0.004
7	0	7	←	6	0	6	5969.1262	0.004
4	2	3	←	3	2	2	3419.097	0.009
4	1	3	←	3	1	2	3452.7966	-0.003
6	3	4	←	5	3	3	5130.2356	-0.007
6	3	3	←	5	3	2	5130.3851	0
6	1	5	←	5	1	4	5177.7682	0.001

Table S20: Measured rotational transitions and residuals (in MHz) of (Benzene)$_2$-$H_2{}^{18}O$ Trimer.

J’	Ka’	Kc’	←	J’’	Ka’’	Kc’’	υ_{obs}	υ_{obs}-υ_{cal}
5	1	5	←	4	1	4	4220.6116	-0.005
5	0	5	←	4	0	4	4268.7958	0.002
5	2	4	←	4	2	3	4277.0436	0.004
5	2	3	←	4	2	2	4286.3375	0.002
5	1	4	←	4	1	3	4331.4458	-0.014
6	1	6	←	5	1	5	5063.5118	-0.003
6	2	5	←	5	2	4	5131.5378	-0.019
6	2	4	←	5	2	3	5147.7169	0.008
6	1	5	←	5	1	4	5196.3347	-0.003
7	1	7	←	6	1	6	5905.7906	-0.007
7	0	7	←	6	0	6	5963.7093	0.012
7	2	6	←	6	2	5	5985.5901	-0.002
7	2	5	←	6	2	4	6011.1381	0.004
7	1	6	←	6	1	5	6060.3858	0
8	1	8	←	7	1	7	6747.4169	0.01
8	0	8	←	7	0	7	6807.127	0.004
3	0	3	←	2	0	2	2565.0037	-0.002
4	1	4	←	3	1	3	3377.1783	-0.001
4	0	4	←	3	0	3	3417.8263	0.004
4	1	3	←	3	1	2	3465.9201	-0.001
3	3	0	←	2	2	1	6513.785	-0.003
3	3	1	←	2	2	0	6513.3059	-0.008
4	3	2	←	3	2	1	7367.9656	0.004
4	2	3	←	4	1	4	2480.0069	0.011

Table S21: Measured rotational transitions and residuals (in MHz) of (Benzene)$_2$-(water)$_2$.

J’	Ka’	Kc’	v’	←	J’’	Ka’’	Kc’’	v’’	υ_{obs}	υ_{obs}-υ_{cal}
3	1	3	0	←	2	1	2	0	2203.0892	-0.005
3	0	3	0	←	2	0	2	0	2282.2416	-0.003

3	1	2	0	←	2	1	1	0	2436.9046	-0.001
4	1	4	0	←	3	1	3	0	2926.0845	-0.005
4	0	4	0	←	3	0	3	0	2999.0968	-0.004
4	2	3	0	←	3	2	2	0	3093.8551	-0.002
4	2	2	0	←	3	2	1	0	3197.8753	0.003
4	1	3	0	←	3	1	2	0	3232.7825	-0.002
5	1	5	0	←	4	1	4	0	3642.0526	-0.004
5	0	5	0	←	4	0	4	0	3696.9489	-0.004
5	1	4	0	←	4	1	3	0	4011.4441	-0.004
6	1	6	0	←	5	1	5	0	4351.8010	-0.004
6	0	6	0	←	5	0	5	0	4387.0602	-0.007
6	2	5	0	←	5	2	4	0	4602.5393	-0.005
6	1	5	0	←	5	1	4	0	4766.0959	0.003
6	2	4	0	←	5	2	3	0	4867.0829	-0.004
7	1	7	0	←	6	1	6	0	5056.7174	-0.009
7	0	7	0	←	6	0	6	0	5077.0195	-0.004
5	2	4	0	←	4	2	3	0	3852.7653	-0.004
5	3	3	0	←	4	3	2	0	3908.3365	-0.010
5	2	3	0	←	4	2	2	0	4034.0713	-0.003
7	2	6	0	←	6	2	5	0	5342.2971	-0.005
7	3	5	0	←	6	3	4	0	5472.1588	-0.003
7	2	5	0	←	6	2	4	0	5685.7279	-0.003
8	1	8	0	←	7	1	7	0	5758.2956	-0.001
8	0	8	0	←	7	0	7	0	5769.1599	0.001
8	2	7	0	←	7	2	6	0	6071.9540	-0.005
8	3	6	0	←	7	3	5	0	6245.0952	-0.007
8	4	5	0	←	7	4	4	0	6276.5925	0.002
5	3	2	0	←	4	3	1	0	3930.5502	0.011
6	3	4	0	←	5	3	3	0	4692.2980	0.000
6	4	2	0	←	5	4	1	0	4695.3021	-0.007
6	3	3	0	←	5	3	2	0	4747.8990	-0.004
7	6	2	0	←	6	6	1	0	5459.9576	0.007
7	6	1	0	←	6	6	0	0	5459.9576	0.002
7	4	4	0	←	6	4	3	0	5483.5798	0.008
7	3	4	0	←	6	3	3	0	5584.5563	0.003
8	5	4	0	←	7	5	3	0	6260.8586	0.011
8	5	3	0	←	7	5	2	0	6262.1472	-0.003
8	4	4	0	←	7	4	3	0	6302.4502	-0.005
8	3	5	0	←	7	3	4	0	6435.9031	0.009
9	1	9	0	←	8	1	8	0	6457.7400	0.000
9	0	9	0	←	8	0	8	0	6463.2727	0.000
8	2	6	0	←	7	2	5	0	6482.8216	-0.008
9	2	8	0	←	8	2	7	0	6792.3042	0.000
9	1	8	0	←	8	1	7	0	6878.3173	0.016
9	3	7	0	←	8	3	6	0	7008.7471	-0.007
9	5	5	0	←	8	5	4	0	7055.1650	-0.004
9	5	4	0	←	8	5	3	0	7059.2996	0.002
9	4	6	0	←	8	4	5	0	7069.3061	-0.001
9	4	5	0	←	8	4	4	0	7126.9556	0.003

10	1	10	0	←	9	1	9	0	7155.9155	0.005
10	0	10	0	←	9	0	9	0	7158.6329	0.002
9	2	7	0	←	8	2	6	0	7252.7163	-0.003
9	3	6	0	←	8	3	5	0	7289.2955	0.009
10	2	9	0	←	9	2	8	0	7504.8153	-0.005
10	3	8	0	←	9	3	7	0	7761.5213	-0.008
11	0	11	0	←	10	0	10	0	7854.6520	0.009
10	4	7	0	←	9	4	6	0	7859.0277	0.010
4	1	4	0	←	3	0	3	0	3131.0932	-0.009
5	0	5	0	←	4	1	4	0	3564.9461	-0.006
3	2	1	0	←	2	1	2	0	3671.3728	0.024
5	1	5	0	←	4	0	4	0	3774.0638	0.005
4	2	3	0	←	3	1	2	0	4036.5216	0.011
3	3	1	0	←	2	2	0	0	4271.7530	-0.020
6	0	6	0	←	5	1	5	0	4309.9581	-0.005
7	0	7	0	←	6	1	6	0	5035.1764	-0.004
7	1	7	0	←	6	0	6	0	5098.5839	0.014
8	0	8	0	←	7	1	7	0	5747.6106	-0.002
8	1	8	0	←	7	0	7	0	5779.8491	0.005
7	2	6	0	←	6	1	5	0	5823.8000	-0.001
8	1	7	0	←	7	2	6	0	5861.5118	-0.006
9	0	9	0	←	8	1	8	0	6452.5867	-0.000
9	1	9	0	←	8	0	8	0	6468.4290	0.005
10	0	10	0	←	9	1	9	0	7153.4770	-0.001
10	1	10	0	←	9	0	9	0	7161.0694	0.007
10	1	9	0	←	9	2	8	0	7435.2978	0.007
3	1	3	1	←	2	1	2	1	2202.8715	-0.002
3	0	3	1	←	2	0	2	1	2282.0405	0.005
3	1	2	1	←	2	1	1	1	2436.8411	0.000
4	1	4	1	←	3	1	3	1	2925.7724	-0.003
4	0	4	1	←	3	0	3	1	2998.7649	0.005
4	2	3	1	←	3	2	2	1	3093.6705	0.001
4	1	3	1	←	3	1	2	1	3232.6711	0.006
5	1	5	1	←	4	1	4	1	3641.6401	-0.003
5	0	5	1	←	4	0	4	1	3696.4793	-0.002
4	2	2	1	←	3	2	1	1	3197.8311	-0.020
5	2	4	1	←	4	2	3	1	3852.5083	0.001
5	3	3	1	←	4	3	2	1	3908.1877	0.009
5	3	2	1	←	4	3	1	1	3930.4333	-0.003
5	1	4	1	←	4	1	3	1	4011.2432	0.009
6	1	6	1	←	5	1	5	1	4351.2825	-0.003
6	0	6	1	←	5	0	5	1	4386.4909	0.004
6	2	5	1	←	5	2	4	1	4602.1940	0.001
6	4	3	1	←	5	4	2	1	4692.0897	0.010
6	3	4	1	←	5	3	3	1	4692.0897	0.003
6	4	2	1	←	5	4	1	1	4695.1288	-0.007
6	3	3	1	←	5	3	2	1	4747.8489	-0.000
6	1	5	1	←	5	1	4	1	4765.7534	0.018
7	1	7	1	←	6	1	6	1	5056.1004	-0.000

7	0	7	1	←	6	0	6	1	5076.3528	0.004
7	2	6	1	←	6	2	5	1	5341.8505	0.006
7	3	5	1	←	6	3	4	1	5471.8969	0.002
7	4	4	1	←	6	4	3	1	5483.3788	0.013
7	2	5	1	←	6	2	4	1	5685.6152	0.006
8	1	8	1	←	7	1	7	1	5757.5695	0.002
8	0	8	1	←	7	0	7	1	5768.3990	0.004
8	2	7	1	←	7	2	6	1	6071.3905	0.005
8	3	6	1	←	7	3	5	1	6244.7583	0.001
8	4	5	1	←	7	4	4	1	6276.3684	0.006
8	4	4	1	←	7	4	3	1	6302.3235	-0.008
9	1	9	1	←	8	1	8	1	6456.9092	0.002
9	0	9	1	←	8	0	8	1	6462.4214	0.003
8	2	6	1	←	7	2	5	1	6482.5833	0.011
9	2	8	1	←	8	2	7	1	6791.6087	0.002
9	3	7	1	←	8	3	6	1	7008.3153	0.005
9	4	6	1	←	8	4	5	1	7069.0416	-0.001
10	1	10	1	←	9	1	9	1	7154.9801	0.003
10	0	10	1	←	9	0	9	1	7157.6938	0.008
9	2	7	1	←	8	2	6	1	7252.2613	-0.006
10	5	6	1	←	9	5	5	1	7852.2237	-0.041
11	1	11	1	←	10	1	10	1	7852.2237	-0.083
5	0	5	1	←	4	1	4	1	3564.7116	-0.018
5	1	5	1	←	4	0	4	1	3773.3909	-0.004
6	0	6	1	←	5	1	5	1	4309.5587	-0.014
7	0	7	1	←	6	1	6	1	5034.6565	0.021
8	0	8	1	←	7	1	7	1	5746.9240	-0.004
8	1	8	1	←	7	0	7	1	5779.0280	-0.004
9	0	9	1	←	8	1	8	1	6451.7794	-0.000
9	1	9	1	←	8	0	8	1	6467.5521	0.007
5	4	1	1	←	4	3	2	1	6613.5445	-0.007
10	0	10	1	←	9	1	9	1	7152.5579	-0.000
10	1	10	1	←	9	0	9	1	7160.1163	0.013
10	1	9	1	←	9	2	8	1	7434.7655	-0.009
10	2	9	1	←	9	1	8	1	7627.9880	0.008

Table S22: Measured rotational transitions and residuals (in MHz) of $(Benzene)_2$-$H_2{}^{18}O(1)$-H_2O.

J’	Ka’	Kc’	←	J’’	Ka’’	Kc’’	υ_{obs}	υ_{obs}-υ_{cal}
3	1	3	←	2	1	2	2191.9971	0.001
4	1	4	←	3	1	3	2911.2214	-0.002
4	0	4	←	3	0	3	2982.7003	-0.000
4	2	3	←	3	2	2	3077.9723	0.015
4	1	3	←	3	1	2	3215.5588	-0.012
5	1	5	←	4	1	4	3623.4251	0.000
5	0	5	←	4	0	4	3676.6941	0.003
5	2	4	←	4	2	3	3832.7822	0.014
6	1	6	←	5	1	5	4329.4423	-0.001
6	0	6	←	5	0	5	4363.3471	0.011

6	2	5	←	5	2	4	4578.3937	0.012
3	0	3	←	2	0	2	2270.0271	-0.001
4	3	1	←	3	3	0	3114.1938	-0.008
4	2	2	←	3	2	1	3182.5376	-0.011
6	5	2	←	5	5	1	4658.6784	-0.000
6	5	1	←	5	5	0	4658.7593	0.015
6	3	3	←	5	3	2	4725.3956	0.017
6	1	5	←	5	1	4	4739.2315	-0.018
7	1	7	←	6	1	6	5030.7031	0.000
7	0	7	←	6	0	6	5050.0311	0.000
7	2	6	←	6	2	5	5313.9586	0.020
7	3	4	←	6	3	3	5558.6312	0.016
7	2	5	←	6	2	4	5656.2591	-0.022
8	1	8	←	7	1	7	5728.6764	-0.005
8	0	8	←	7	0	7	5738.9336	-0.000
8	2	7	←	7	2	6	6039.4281	0.026
8	3	5	←	7	3	4	6406.0242	-0.005
9	1	9	←	8	1	8	6424.5816	-0.014
9	0	9	←	8	0	8	6429.7852	0.010
8	2	6	←	7	2	5	6447.8461	-0.016
10	1	10	←	9	1	9	7119.2711	-0.015
4	0	4	←	3	1	3	2784.5556	-0.003
4	1	4	←	3	0	3	3109.3608	-0.004
5	0	5	←	4	1	4	3550.0049	-0.020
5	1	5	←	4	0	4	3750.0876	-0.002
6	1	6	←	5	0	5	4402.8426	-0.001
8	1	8	←	7	0	7	5748.8656	0.004
9	1	9	←	8	0	8	6434.5280	0.004
10	1	10	←	9	0	9	7124.0298	-0.006

Table S23: Measured rotational transitions and residuals (in MHz) of $(Benzene)_2$-H_2O-$H_2{}^{18}O$ (2).

J'	Ka'	Kc'	←	J''	Ka''	Kc''	υ_{obs}	υ_{obs}-υ_{cal}
3	1	2	←	2	1	1	2425.5639	0.006
4	1	4	←	3	1	3	2884.8971	0.005
4	0	4	←	3	0	3	2953.4312	0.011
4	2	2	←	3	2	1	3192.1423	-0.012
4	1	3	←	3	1	2	3213.1051	-0.000
5	1	5	←	4	1	4	3587.8088	0.010
5	0	5	←	4	0	4	3635.1567	0.005
5	2	4	←	4	2	3	3815.7737	-0.017
5	1	4	←	4	1	3	3978.3977	0.000
5	2	3	←	4	2	2	4027.7337	-0.015
6	1	6	←	5	1	5	4284.0285	0.008
3	0	3	←	2	0	2	2253.4742	-0.000
4	2	3	←	3	2	2	3067.0464	-0.019
6	0	6	←	5	0	5	4311.8700	0.003
6	1	5	←	5	1	4	4713.4379	-0.003
6	2	4	←	5	2	3	4854.3182	-0.011

7	1	7	←	6	1	6	4975.4213	0.008
7	0	7	←	6	0	6	4990.1312	-0.002
7	2	5	←	6	2	4	5660.2791	-0.003
8	1	8	←	7	1	7	5663.7106	0.003
8	0	8	←	7	0	7	5670.9646	-0.006
7	1	6	←	6	1	5	5415.8579	0.002
9	1	9	←	8	1	8	6350.1677	-0.015
9	0	9	←	8	0	8	6353.5873	-0.017
9	3	6	←	8	3	5	7283.6066	0.026
6	1	6	←	5	0	5	4340.0551	0.007
7	0	7	←	6	1	6	4961.9677	0.015
7	1	7	←	6	0	6	5003.5944	0.000
8	0	8	←	7	1	7	5657.5178	0.007
8	1	8	←	7	0	7	5677.1610	-0.007
9	0	9	←	8	1	8	6347.4026	-0.004
9	1	9	←	8	0	8	6356.4007	0.020
10	1	10	←	9	0	9	7038.4109	-0.010
10	1	9	←	9	2	8	7355.3720	0.000

Table S24: Measured rotational transitions and residuals (in MHz) of $(Benzene)_3$-water.

J’	Ka’	Kc’	←	J’’	Ka’’	Kc’’	υ_{obs}	υ_{obs}-υ_{cal}
4	2	2	←	3	2	1	2172.8898	-0.001
5	1	5	←	4	1	4	2213.7003	0.002
5	0	5	←	4	0	4	2217.7506	0.003
5	2	4	←	4	2	3	2428.8979	0.004
5	3	3	←	4	3	2	2554.8133	-0.000
6	1	6	←	5	1	5	2633.8007	0.001
6	0	6	←	5	0	5	2634.9300	-0.001
5	3	2	←	4	3	1	2688.0881	-0.001
5	2	3	←	4	2	2	2697.9079	-0.001
6	2	5	←	5	2	4	2865.9925	0.005
6	1	5	←	5	1	4	2894.4501	0.001
6	3	4	←	5	3	3	3038.5552	0.003
7	1	7	←	6	1	6	3052.9473	0.001
7	0	7	←	6	0	6	3053.2378	-0.001
6	5	2	←	5	5	1	3096.7479	0.010
6	4	3	←	5	4	2	3102.9469	-0.002
6	4	2	←	5	4	1	3167.5053	-0.002
6	2	4	←	5	2	3	3171.4301	0.004
6	3	3	←	5	3	2	3265.1646	-0.003
7	2	6	←	6	2	5	3292.5739	0.000
7	1	6	←	6	1	5	3302.9815	0.000
8	1	8	←	7	1	7	3471.8210	-0.001
8	0	8	←	7	0	7	3471.8785	-0.016
7	2	5	←	6	2	4	3592.2840	0.001
7	5	3	←	6	5	2	3629.7851	0.000
7	5	2	←	6	5	1	3653.0482	-0.000
5	1	4	←	4	1	3	2489.9713	0.003

7	4	4	←	6	4	3	3614.6297	0.002
7	3	5	←	6	3	4	3500.5257	0.003
6	5	1	←	5	5	0	3101.5229	-0.016
8	2	7	←	7	2	6	3714.0193	-0.001
8	1	7	←	7	1	6	3717.3196	-0.004
7	4	3	←	6	4	2	3766.3592	-0.001
7	3	4	←	6	3	3	3801.2763	0.002
9	1	9	←	8	1	8	3890.6376	0.004
9	0	9	←	8	0	8	3890.6376	-0.012
8	3	6	←	7	3	5	3943.2885	-0.025
8	2	6	←	7	2	5	3989.7936	-0.000
8	4	5	←	7	4	4	4106.5370	0.003
9	2	8	←	8	2	7	4133.4855	-0.001
9	1	8	←	8	1	7	4134.4415	-0.005
8	6	3	←	7	6	2	4146.0452	0.001
8	6	2	←	7	6	1	4152.9942	0.004
8	5	4	←	7	5	3	4160.3583	0.000
8	5	3	←	7	5	2	4234.7755	-0.000
8	3	5	←	7	3	4	4279.2023	0.003
10	1	10	←	9	1	9	4309.4256	-0.003
10	0	10	←	9	0	9	4309.4256	-0.007
8	4	4	←	7	4	3	4358.5887	-0.000
9	3	7	←	8	3	6	4372.9462	-0.001
9	2	7	←	8	2	6	4391.5996	-0.003
6	4	2	←	5	2	3	4437.5032	-0.008
10	2	9	←	9	2	8	4552.3361	0.001
10	1	9	←	9	1	8	4552.5943	-0.003
9	4	6	←	8	4	5	4575.2520	0.002
9	5	5	←	8	5	4	4679.1940	0.006
9	6	4	←	8	6	3	4685.3236	0.001
9	3	6	←	8	3	5	4698.6071	-0.001
9	6	3	←	8	6	2	4713.2234	-0.000
11	1	11	←	10	1	10	4728.2182	-0.003
11	0	11	←	10	0	10	4728.2182	-0.004
10	3	8	←	9	3	7	4795.5466	-0.000
10	2	8	←	9	2	7	4802.0166	-0.002
9	4	5	←	8	4	4	4906.0129	0.004
10	4	7	←	9	4	6	5022.7956	-0.000
7	4	3	←	6	2	4	5032.4357	-0.011
10	5	6	←	9	5	5	5177.9126	0.002
10	7	4	←	9	7	3	5199.8673	0.007
10	7	3	←	9	7	2	5208.6563	0.007
11	3	9	←	10	3	8	5215.1807	-0.000
11	2	9	←	10	2	8	5217.2261	0.000
10	6	5	←	9	6	4	5222.0148	0.003
9	5	4	←	8	5	3	4845.9301	-0.001
11	2	10	←	10	2	9	4971.0457	0.008
10	6	4	←	9	6	3	5304.1130	0.000
10	4	6	←	9	4	5	5389.4129	0.001

12	2	11	←	11	2	10	5389.7363	0.010
12	1	11	←	11	1	10	5389.7363	-0.006
10	5	5	←	9	5	4	5452.1979	-0.000
11	4	8	←	10	4	7	5455.0685	0.002
11	3	8	←	10	3	7	5483.7206	-0.002
13	0	13	←	12	0	12	5565.8030	0.001
13	1	13	←	12	1	12	5565.8030	0.001
12	3	10	←	11	3	9	5633.8373	0.003
12	2	10	←	11	2	9	5634.4374	-0.001
11	5	7	←	10	5	6	5652.4479	0.001
11	7	5	←	10	7	4	5744.5784	0.001
11	6	6	←	10	6	5	5747.0401	0.001
11	7	4	←	10	7	3	5776.1616	0.000
11	4	7	←	10	4	6	5808.3524	-0.006
13	2	12	←	12	2	11	5808.4317	0.003
13	1	12	←	12	1	11	5808.4317	-0.000
12	4	9	←	11	4	8	5878.6300	0.000
12	3	9	←	11	3	8	5889.2711	-0.003
11	6	5	←	10	6	4	5925.9303	-0.004
14	1	14	←	13	1	13	5984.5867	0.000
14	0	14	←	13	0	13	5984.5867	0.000
11	5	6	←	10	5	5	6011.3752	0.003
13	3	11	←	12	3	10	6052.2755	0.010
13	2	11	←	12	2	10	6052.4321	-0.003
12	5	8	←	11	5	7	6104.2275	0.006
12	4	8	←	11	4	7	6192.7693	-0.001
14	2	13	←	13	2	12	6227.1437	-0.001
14	1	13	←	13	1	12	6227.1437	-0.002
12	6	7	←	11	6	6	6251.8025	0.002
12	8	5	←	11	8	4	6257.0193	-0.000
12	8	4	←	11	8	3	6267.3545	-0.001
12	7	6	←	11	7	5	6286.6866	-0.002
13	4	10	←	12	4	9	6298.2843	0.009
13	3	10	←	12	3	9	6301.8651	-0.000
12	7	5	←	11	7	4	6374.7296	-0.000
14	2	12	←	13	2	11	6470.7484	-0.003
12	5	7	←	11	5	6	6501.3858	0.002
13	5	9	←	12	5	8	6538.8822	-0.002
12	6	6	←	11	6	5	6545.4331	0.000
13	4	9	←	12	4	8	6579.2579	-0.000
15	2	14	←	14	2	13	6645.8730	0.001
15	1	14	←	14	1	13	6645.8730	0.001
14	3	11	←	13	3	10	6717.6909	0.000
13	6	8	←	12	6	7	6731.6328	0.006
13	8	6	←	12	8	5	6806.5950	0.005
13	7	7	←	12	7	6	6817.3025	0.001
13	8	5	←	12	8	4	6841.0693	0.007
15	3	13	←	14	3	12	6889.1948	-0.008
13	5	8	←	12	5	7	6920.9998	-0.000

14	5	10	←	13	5	9	6963.2955	0.003
14	4	10	←	13	4	9	6979.1466	0.000
13	6	7	←	12	6	6	7116.8353	0.002
15	4	12	←	14	4	11	7134.5483	0.002
15	3	12	←	14	3	11	7134.8826	-0.002
14	6	9	←	13	6	8	7187.3325	-0.001
14	5	9	←	13	5	8	7299.7423	-0.002
14	7	8	←	13	7	7	7327.6116	-0.001
7	6	2	←	6	5	2	5042.0087	0.000
7	6	1	←	6	5	1	5037.9748	0.005
3	3	0	←	2	2	0	2186.1427	0.000
3	3	1	←	2	2	1	2210.5305	-0.006
4	2	2	←	3	1	2	2513.9246	-0.000
4	1	3	←	3	0	3	2567.3428	-0.001
4	2	3	←	3	1	3	2653.6131	-0.003
4	3	1	←	3	2	1	2676.9435	0.001
4	3	2	←	3	2	2	2759.9675	-0.001
4	4	0	←	3	3	0	2970.6567	0.000
4	4	1	←	3	3	1	2978.2925	-0.000
5	2	3	←	4	1	3	3144.8230	-0.001
5	3	2	←	4	2	2	3192.1397	-0.000
5	1	4	←	4	0	4	3253.8424	0.000
5	2	4	←	4	1	4	3291.7577	-0.001
5	3	3	←	4	2	3	3339.3899	-0.000
5	4	1	←	4	3	1	3463.8646	0.002
5	4	2	←	4	3	2	3505.8544	0.001
5	5	0	←	4	4	0	3750.4218	0.002
5	5	1	←	4	4	1	3752.3041	0.001
6	3	3	←	5	2	3	3759.3972	-0.001
6	2	4	←	5	1	4	3826.2823	-0.000
6	1	5	←	5	0	5	3930.5503	0.006
6	4	2	←	5	3	2	3943.2886	0.007
6	2	5	←	5	1	5	3944.0495	0.000
6	3	4	←	5	2	4	3949.0474	-0.000
6	4	3	←	5	3	3	4053.9893	0.001
6	5	1	←	5	4	1	4255.0005	0.004
6	5	2	←	5	4	2	4269.5203	0.002
7	3	4	←	6	2	4	4389.2440	-0.002
7	4	3	←	6	3	3	4444.4754	0.001
7	2	5	←	6	1	5	4524.1158	-0.001
6	6	0	←	5	5	0	4527.9224	0.004
6	6	1	←	5	5	1	4528.3309	0.001
7	3	5	←	6	2	5	4583.5827	-0.000
7	1	6	←	6	0	6	4598.5957	0.002
7	2	6	←	6	1	6	4602.8141	-0.008
7	4	4	←	6	3	4	4630.0648	0.001
7	5	2	←	6	4	2	4740.5394	0.002
7	5	3	←	6	4	3	4796.3557	0.002
8	4	4	←	7	3	4	5001.7891	-0.000

8	3	5	←	7	2	5	5076.1609	-0.002
8	5	3	←	7	4	3	5208.9532	0.001
8	2	6	←	7	1	6	5210.9315	0.000
8	3	6	←	7	2	6	5234.3204	-0.004
8	4	5	←	7	3	5	5236.0890	0.014
8	1	7	←	7	0	7	5262.6668	-0.011
8	2	7	←	7	1	7	5263.9012	0.003
7	7	0	←	6	6	0	5304.7838	0.011
7	7	1	←	6	6	1	5304.8457	-0.010
8	5	4	←	7	4	4	5342.0857	0.002
8	6	2	←	7	5	2	5537.9111	0.000
8	6	3	←	7	5	3	5558.2679	0.000
9	4	5	←	8	3	5	5628.5960	-0.003
9	5	4	←	8	4	4	5696.2962	0.002
9	3	6	←	8	2	6	5784.9757	-0.001
8	7	1	←	7	6	1	5816.6979	0.002
8	7	2	←	7	6	2	5817.6885	0.000
9	4	6	←	8	3	6	5868.0087	-0.001
9	2	7	←	8	1	7	5885.2086	-0.001
9	3	7	←	8	2	7	5893.2527	0.001
9	5	5	←	8	4	5	5914.7379	0.000
9	1	8	←	8	0	8	5925.2372	0.007
9	6	3	←	8	5	3	6016.3576	-0.000
8	8	0	←	7	7	0	6081.4735	0.016
8	8	1	←	7	7	1	6081.4735	-0.000
9	6	4	←	8	5	4	6083.2321	0.000
10	5	5	←	9	4	5	6242.4827	-0.000
10	4	6	←	9	3	6	6319.3986	-0.003
9	7	2	←	8	6	2	6324.1223	0.003
9	7	3	←	8	6	3	6330.2111	-0.001
10	6	4	←	9	5	4	6474.5375	-0.002
10	3	7	←	9	2	7	6482.7174	-0.000
10	5	6	←	9	4	6	6517.3980	-0.000
10	4	7	←	9	3	7	6517.8561	-0.002
10	3	8	←	9	2	8	6555.3132	0.001
9	8	1	←	8	7	1	6594.0134	0.005
9	8	2	←	8	7	2	6594.2311	-0.001
10	6	5	←	9	5	5	6626.0577	0.002
10	7	3	←	9	6	3	6819.5435	-0.000
10	7	4	←	9	6	4	6844.7471	-0.003
9	9	0	←	8	8	0	6858.0887	0.002
9	9	1	←	8	8	1	6858.0887	-0.000
11	5	6	←	10	4	6	6864.4363	-0.007
11	6	5	←	10	5	5	6948.2755	-0.000
11	4	7	←	10	3	7	7038.4113	-0.006
10	8	2	←	9	7	2	7104.3442	0.005
10	8	3	←	9	7	3	7105.9557	-0.000
11	5	7	←	10	4	7	7147.0450	-0.003
11	3	8	←	10	2	8	7164.4245	0.002

11	4	8	←	10	3	8	7177.3770	-0.000
11	6	6	←	10	5	6	7195.1829	-0.001
11	2	9	←	10	1	9	7217.4194	0.008
11	3	9	←	10	2	9	7218.1586	0.000
11	1	10	←	10	0	10	7248.8713	0.020
11	2	10	←	10	1	10	7248.8713	-0.001
11	7	4	←	10	6	4	7291.5870	-0.005
11	7	5	←	10	6	5	7367.3127	-0.003
10	9	1	←	9	8	1	7370.8720	-0.012
10	9	2	←	9	8	2	7370.9212	-0.011
12	6	6	←	11	5	6	7482.3342	-0.002
12	5	7	←	11	4	7	7557.4621	-0.006
11	8	3	←	10	7	3	7608.9163	0.003
11	8	4	←	10	7	4	7616.8374	-0.007
10	10	0	←	9	9	0	7634.6966	0.011
10	10	1	←	9	9	1	7634.6966	0.010
12	7	5	←	11	6	5	7740.3851	-0.002
12	4	8	←	11	3	8	7747.4662	0.000
12	6	7	←	11	5	7	7794.5312	-0.006

Table S25: Measured rotational transitions and residuals (in MHz) of (Benzene)$_3$-$H_2{}^{18}O$.

J’	Ka’	Kc’	←	J’’	Ka’’	Kc’’	υ_{obs}	υ_{obs}-υ_{cal}
3	3	1	←	2	2	1	2189.9336	-0.001
4	2	2	←	3	1	2	2494.3908	-0.001
4	1	3	←	3	0	3	2546.5603	-0.001
4	2	3	←	3	1	3	2631.4520	-0.004
4	3	1	←	3	2	1	2654.6841	-0.005
4	3	2	←	3	2	2	2735.9777	0.000
4	4	0	←	3	3	0	2942.8219	0.002
4	4	1	←	3	3	1	2950.2702	-0.000
5	2	3	←	4	1	3	3120.1264	0.000
5	3	2	←	4	2	2	3167.0504	0.000
5	1	4	←	4	0	4	3227.0597	-0.004
5	2	4	←	4	1	4	3264.4532	-0.002
5	3	3	←	4	2	3	3311.3684	-0.003
5	4	1	←	4	3	1	3433.8892	0.002
5	4	2	←	4	3	2	3474.8964	0.000
5	5	0	←	4	4	0	3714.8978	0.001
5	5	1	←	4	4	1	3716.7306	0.000
6	3	3	←	5	2	3	3730.3218	-0.002
6	2	4	←	5	1	4	3795.4798	-0.002
6	1	5	←	5	0	5	3898.0047	-0.000
6	4	2	←	5	3	2	3911.3461	0.001
6	2	5	←	5	1	5	3911.3461	-0.004
6	3	4	←	5	2	4	3916.3815	-0.004
6	4	3	←	5	3	3	4019.6281	0.003
6	5	1	←	5	4	1	4217.1012	0.001
6	5	2	←	5	4	2	4231.2482	0.001

7	3	4	←	6	2	4	4354.8784	-0.003
7	4	3	←	6	3	3	4409.8915	0.002
6	6	0	←	5	5	0	4484.7646	0.004
6	6	1	←	5	5	1	4485.1597	-0.000
7	2	5	←	6	1	5	4487.0978	0.000
7	3	5	←	6	2	5	4545.8017	-0.001
7	1	6	←	6	0	6	4560.4469	0.004
7	2	6	←	6	1	6	4564.6250	-0.004
7	4	4	←	6	3	4	4591.6864	0.000
7	5	2	←	6	4	2	4700.6977	-0.000
7	5	3	←	6	4	3	4755.1385	0.002
8	4	4	←	7	3	4	4963.2639	0.003
7	6	1	←	6	5	1	4992.3070	0.003
7	6	2	←	6	5	2	4996.2277	-0.001
8	3	5	←	7	2	5	5035.4775	-0.000
8	2	6	←	7	1	6	5167.9985	-0.002
8	3	6	←	7	2	6	5191.1427	-0.005
8	4	5	←	7	3	5	5193.0690	0.000
8	1	7	←	7	0	7	5218.9805	0.010
7	7	0	←	6	6	0	5253.9997	0.005
7	7	1	←	6	6	1	5254.0601	-0.015
8	5	4	←	7	4	4	5297.4696	0.001
8	6	2	←	7	5	2	5489.9976	0.000
8	6	3	←	7	5	3	5509.7979	0.000
9	4	5	←	8	3	5	5584.6682	-0.000
9	5	4	←	8	4	4	5652.2730	-0.000
9	3	6	←	8	2	6	5737.8381	-0.002
8	7	1	←	7	6	1	5763.3609	0.000
8	7	2	←	7	6	2	5764.3170	-0.005
9	4	6	←	8	3	6	5819.8901	0.002
9	2	7	←	8	1	7	5836.5903	0.003
9	3	7	←	8	2	7	5844.5562	-0.004
9	5	5	←	8	4	5	5866.1257	0.004
9	6	3	←	8	5	3	5966.6949	-0.001
8	8	0	←	7	7	0	6023.0760	0.013
8	8	1	←	7	7	1	6023.0760	-0.002
9	6	4	←	8	5	4	6031.8319	0.001
10	5	5	←	9	4	5	6194.5696	0.000
9	7	2	←	8	6	2	6268.3435	-0.002
10	4	6	←	9	3	6	6268.9311	-0.002
9	7	3	←	8	6	3	6274.2533	-0.005
10	6	4	←	9	5	4	6423.3888	-0.001
10	3	7	←	9	2	7	6429.5092	-0.000
10	5	6	←	9	4	6	6464.1694	0.008
10	4	7	←	9	3	7	6464.3200	-0.001
10	2	8	←	9	1	8	6498.5609	0.002
9	8	1	←	8	7	1	6533.0419	0.003
9	8	2	←	8	7	2	6533.2594	0.002
10	6	5	←	9	5	5	6571.2754	0.000

10	7	3	←	9	6	3	6761.6446	-0.006
10	7	4	←	9	6	4	6786.1265	-0.005
9	9	0	←	8	8	0	6792.0812	0.004
9	9	1	←	8	8	1	6792.0812	0.002
11	5	6	←	10	4	6	6811.0265	-0.000
11	6	5	←	10	5	5	6894.8706	-0.000
11	4	7	←	10	3	7	6981.2553	-0.001
10	8	2	←	9	7	2	7040.8588	0.006
10	8	3	←	9	7	3	7042.4273	0.008
11	5	7	←	10	4	7	7088.6826	-0.001
11	3	8	←	10	2	8	7105.4355	0.005
11	4	8	←	10	3	8	7118.2965	0.002
11	6	6	←	10	5	6	7136.4145	-0.004
11	1	10	←	10	0	10	7188.5608	0.010
11	2	10	←	10	1	10	7188.5608	-0.010
11	7	4	←	10	6	4	7232.1307	-0.006
10	9	1	←	9	8	1	7302.2901	-0.003
10	9	2	←	9	8	2	7302.3362	-0.003
11	7	5	←	10	6	5	7305.7885	-0.004
12	6	6	←	11	5	6	7425.0955	-0.001
5	1	5	←	4	1	4	2208.4096	0.000
5	0	5	←	4	0	4	2212.4068	0.004
5	2	4	←	4	2	3	2419.1022	0.005
5	1	4	←	4	1	3	2479.1189	0.003
6	1	6	←	5	1	5	2627.9231	-0.002
6	0	6	←	5	0	5	2629.0405	-0.003
5	3	2	←	4	3	1	2672.3910	-0.000
5	2	3	←	4	2	2	2682.4047	0.000
6	2	5	←	5	2	4	2855.3067	0.002
6	1	5	←	5	1	4	2883.3459	0.003
6	3	4	←	5	3	3	3024.1141	0.003
7	1	7	←	6	1	6	3046.5013	-0.000
7	0	7	←	6	0	6	3046.7832	-0.008
6	4	3	←	5	4	2	3086.9467	-0.000
6	4	2	←	5	4	1	3149.8488	-0.000
6	2	4	←	5	2	3	3154.4742	0.002
6	3	3	←	5	3	2	3245.6814	0.002
7	2	6	←	6	2	5	3281.2040	-0.000
7	1	6	←	6	1	5	3291.4793	-0.001
7	3	5	←	6	3	4	3484.7277	0.006
7	2	5	←	6	2	4	3574.9574	-0.000
7	4	4	←	6	4	3	3596.1753	0.003
7	5	3	←	6	5	2	3610.8427	0.005
7	5	2	←	6	5	1	3633.4457	-0.002
8	2	7	←	7	2	6	3702.0509	-0.001
8	1	7	←	7	1	6	3705.3172	-0.003
7	4	3	←	6	4	2	3744.2219	-0.001
7	3	4	←	6	3	3	3779.0324	0.002
9	1	9	←	8	1	8	3883.0565	0.001

9	0	9	←	8	0	8	3883.0565	-0.015
8	3	6	←	7	3	5	3926.5525	0.002
8	2	6	←	7	2	5	3972.3814	-0.002
8	4	5	←	7	4	4	4086.1062	0.001
9	2	8	←	8	2	7	4120.9434	-0.002
9	1	8	←	8	1	7	4121.8964	-0.001
8	5	4	←	7	5	3	4138.5021	-0.001
8	5	3	←	7	5	2	4210.9158	0.001
8	3	5	←	7	3	4	4255.5609	0.006
10	1	10	←	9	1	9	4301.2838	-0.000
10	0	10	←	9	0	9	4301.2838	-0.004
8	4	4	←	7	4	3	4332.4021	0.001
9	3	7	←	8	3	6	4355.4621	-0.002
9	2	7	←	8	2	6	4373.9038	-0.002
10	2	9	←	9	2	8	4539.2341	0.005
10	1	9	←	9	1	8	4539.4758	-0.014
9	4	6	←	8	4	5	4553.3564	-0.011
9	5	5	←	8	5	4	4654.7586	0.000
9	6	4	←	8	6	3	4660.5434	0.006
9	3	6	←	8	3	5	4674.7453	-0.000
11	1	11	←	10	1	10	4719.5077	-0.003
11	0	11	←	10	0	10	4719.5077	-0.004
10	3	8	←	9	3	7	4777.4587	0.002
10	2	8	←	9	2	7	4783.8652	-0.003
9	5	4	←	8	5	3	4817.2855	-0.001
9	4	5	←	8	4	4	4876.9664	0.003
11	2	10	←	10	2	9	4957.3736	0.006
11	1	10	←	10	1	9	4957.4327	-0.002
10	4	7	←	9	4	6	4999.8979	-0.000
10	3	7	←	9	3	6	5065.5756	0.000
12	1	12	←	11	1	11	5137.7348	-0.001
12	0	12	←	11	0	11	5137.7348	-0.001
10	5	6	←	9	5	5	5151.4084	0.001
10	6	5	←	9	6	4	5194.2026	0.000
11	3	9	←	10	3	8	5196.5237	0.001
11	2	9	←	10	2	8	5198.5568	0.004
10	6	4	←	9	6	3	5273.9767	-0.003
10	4	6	←	9	4	5	5359.0238	0.012
12	2	11	←	11	2	10	5375.5014	0.010
12	1	11	←	11	1	10	5375.5014	-0.006
10	5	5	←	9	5	4	5419.2565	-0.000
11	4	8	←	10	4	7	5431.4255	-0.003
11	3	8	←	10	3	7	5459.7847	-0.004
13	1	13	←	12	1	12	5555.9547	-0.003
13	0	13	←	12	0	12	5555.9547	-0.003
12	2	10	←	11	2	9	5615.2268	0.008
11	5	7	←	10	5	6	5624.4281	0.006
11	4	7	←	10	4	6	5777.9086	0.010
13	2	12	←	12	2	11	5793.6299	0.001

13	1	12	←	12	1	11	5793.6299	-0.002
12	4	9	←	11	4	8	5854.3846	-0.002
11	6	5	←	10	6	4	5890.7436	0.005
14	0	14	←	13	0	13	5974.1741	-0.002
14	1	14	←	13	1	13	5974.1741	-0.002
12	5	8	←	11	5	7	6075.1491	0.007
12	4	8	←	11	4	7	6162.5951	-0.002
14	2	13	←	13	2	12	6211.7822	0.002
14	1	13	←	13	1	12	6211.7822	0.001
13	3	10	←	12	3	9	6277.0414	-0.001
12	7	5	←	11	7	4	6338.2751	-0.006
15	0	15	←	14	0	14	6392.3859	-0.004
15	1	15	←	14	1	14	6392.3859	-0.004
12	5	7	←	11	5	6	6464.1694	-0.000
13	5	9	←	12	5	8	6509.0558	-0.000
15	2	14	←	14	2	13	6629.9478	0.007
15	1	14	←	14	1	13	6629.9478	0.007
13	5	8	←	12	5	7	6883.8838	0.000
14	5	10	←	13	5	9	6932.8704	0.004
13	7	6	←	12	7	5	6964.1146	-0.005
14	5	9	←	13	5	8	7263.1190	-0.001
14	7	8	←	13	7	7	7288.7737	-0.001

Table S26: Measured rotational transitions and residuals (in MHz) of (Benzene)$_3$-(H_2O)$_2$.

J’	Ka’	Kc’	←	J’’	Ka’’	Kc’’	υ_{obs}	υ_{obs}-υ_{cal}
4	3	2	←	3	2	2	2375.7217	0.009
5	2	3	←	4	1	3	2569.7011	0.002
4	4	0	←	3	3	0	2605.8730	-0.005
5	2	4	←	4	1	4	2724.1900	-0.017
5	3	3	←	4	2	3	2835.0899	0.005
5	4	1	←	4	3	1	3039.3157	0.000
5	4	2	←	4	3	2	3046.1446	0.000
6	2	4	←	5	1	4	3065.9096	0.006
6	3	4	←	5	2	4	3308.4613	0.006
6	4	2	←	5	3	2	3464.7071	-0.001
6	4	3	←	5	3	3	3489.0642	-0.001
7	2	5	←	6	1	5	3593.6113	0.005
7	3	4	←	6	2	4	3616.7980	0.003
7	3	5	←	6	2	5	3797.2197	0.000
7	4	3	←	6	3	3	3878.0684	-0.002
7	4	4	←	6	3	4	3938.9729	0.001
6	6	0	←	5	5	0	3970.9377	-0.008
6	6	1	←	5	5	1	3970.9775	0.018
8	3	5	←	7	2	5	4083.0744	0.004
8	2	6	←	7	1	6	4148.9348	0.008
7	5	2	←	6	4	2	4158.5917	-0.005
7	5	3	←	6	4	3	4163.6239	-0.003
8	4	4	←	7	3	4	4282.9907	-0.001

8	3	6	←	7	2	6	4301.2197	0.001
9	3	6	←	8	2	6	4583.5171	0.009
8	5	3	←	7	4	3	4585.9457	-0.000
8	5	4	←	7	4	4	4602.6585	0.001
7	7	0	←	6	6	0	4653.1807	-0.003
7	7	1	←	6	6	1	4653.1807	-0.004
9	4	5	←	8	3	5	4693.4220	-0.001
9	2	7	←	8	1	7	4718.8131	0.006
8	6	2	←	7	5	2	4843.8998	0.019
8	6	3	←	7	5	3	4844.6971	-0.000
9	4	6	←	8	3	6	4873.5015	0.001
9	5	4	←	8	4	4	5002.2113	-0.011
9	5	5	←	8	4	5	5045.5227	0.002
8	7	1	←	7	6	1	5090.3051	0.011
8	7	2	←	7	6	2	5090.3051	-0.005
10	3	7	←	9	2	7	5119.5253	0.009
10	4	6	←	9	3	6	5126.0837	-0.002
9	6	3	←	8	5	3	5277.5806	0.003
9	6	4	←	8	5	4	5280.8946	-0.001
10	2	8	←	9	1	8	5288.9406	0.004
8	8	0	←	7	7	0	5335.4043	0.003
8	8	1	←	7	7	1	5335.4043	0.003
10	3	8	←	9	2	8	5347.0752	0.002
10	4	7	←	9	3	7	5362.7633	0.007
10	1	9	←	9	0	9	5383.6382	0.005
10	2	9	←	9	1	9	5388.1119	0.002
10	5	5	←	9	4	5	5404.8152	-0.002
10	5	6	←	9	4	6	5495.5573	0.005
11	4	7	←	10	3	7	5592.3212	0.002
11	3	8	←	10	2	8	5684.7024	0.010
10	6	4	←	9	5	4	5706.5664	0.003
10	6	5	←	9	5	5	5717.1974	-0.001
9	8	1	←	8	7	1	5772.5676	-0.002
9	8	2	←	8	7	2	5772.5676	-0.004
11	5	6	←	10	4	6	5799.6337	-0.000
11	2	9	←	10	1	9	5852.1943	-0.003
11	4	8	←	10	3	8	5867.2583	0.007
11	3	9	←	10	2	9	5882.9127	-0.009
11	5	7	←	10	4	7	5956.3498	0.001
10	7	3	←	9	6	3	5962.4836	0.004
10	7	4	←	9	6	4	5963.0221	-0.005
9	9	0	←	8	8	0	6017.6170	0.013
9	9	1	←	8	8	1	6017.6170	0.013
12	4	8	←	11	3	8	6097.5779	0.002
11	6	5	←	10	5	5	6126.8569	-0.002
11	6	6	←	10	5	6	6155.0950	-0.002
12	5	7	←	11	4	7	6202.6060	-0.002
10	8	2	←	9	7	2	6209.3933	-0.012
10	8	3	←	9	7	3	6209.4243	0.003

12	3	9	←	11	2	9	6264.0501	0.001
12	4	9	←	11	3	9	6385.4221	0.003
11	7	4	←	10	6	4	6396.1194	-0.000
11	7	5	←	10	6	5	6398.1795	-0.001
12	5	8	←	11	4	8	6430.8842	-0.005
10	9	1	←	9	8	1	6454.7978	-0.004
10	9	2	←	9	8	2	6454.7978	-0.005
12	6	6	←	11	5	6	6533.7971	0.001
12	6	7	←	11	5	7	6597.1766	-0.003
13	5	8	←	12	4	8	6631.6799	-0.004
11	8	3	←	10	7	3	6645.6098	-0.009
11	8	4	←	10	7	4	6645.6776	-0.022
10	10	0	←	9	9	0	6699.8020	0.012
10	10	1	←	9	9	1	6699.8020	0.012
12	7	5	←	11	6	5	6826.1938	0.011
12	7	6	←	11	6	6	6832.6447	0.002
13	3	10	←	12	2	10	6842.3668	0.005
11	9	2	←	10	8	2	6891.7492	-0.007
11	9	3	←	10	8	3	6891.7492	-0.009
13	5	9	←	12	4	9	6920.8492	0.003
13	6	7	←	12	5	7	6926.0138	-0.000
13	1	12	←	12	0	12	7018.0141	0.021
13	6	8	←	12	5	8	7046.8726	-0.003
14	5	9	←	13	4	9	7098.8801	0.021
11	10	1	←	10	9	1	7136.9973	-0.010
11	10	2	←	10	9	2	7137.0222	0.014
14	4	10	←	13	3	10	7216.6238	0.012
13	7	6	←	12	6	6	7249.7648	-0.032
13	7	7	←	12	6	7	7267.2143	-0.003
14	6	8	←	13	5	8	7311.4913	-0.012
12	9	3	←	11	8	3	7328.2801	0.012
12	9	4	←	11	8	4	7328.2801	0.001
11	11	0	←	10	10	0	7381.9489	-0.012
11	11	1	←	10	10	1	7381.9782	0.017
14	5	10	←	13	4	10	7426.2512	0.018
14	2	12	←	13	1	12	7507.4963	0.024
14	7	7	←	13	6	7	7662.6380	-0.007
5	1	5	←	4	1	4	2052.8621	0.006
5	0	5	←	4	0	4	2065.7216	-0.003
5	2	4	←	4	2	3	2154.1094	-0.005
5	3	3	←	4	3	2	2190.8243	0.002
5	3	2	←	4	3	1	2213.0906	-0.000
5	1	4	←	4	1	3	2217.2140	-0.004
5	2	3	←	4	2	2	2259.7697	0.002
6	1	6	←	5	1	5	2453.2857	-0.002
6	0	6	←	5	0	5	2459.6473	-0.003
6	2	5	←	5	2	4	2570.8489	-0.002
6	1	5	←	5	1	4	2625.8774	-0.005
6	3	4	←	5	3	3	2627.4812	-0.003

6	4	3	←	5	4	2	2633.7427	-0.000
6	4	2	←	5	4	1	2638.4787	-0.005
6	3	3	←	5	3	2	2678.7228	0.002
6	2	4	←	5	2	3	2713.4249	0.001
7	1	7	←	6	1	6	2852.0450	-0.000
7	0	7	←	6	0	6	2854.8967	-0.002
7	2	6	←	6	2	5	2981.7398	-0.004
7	1	6	←	6	1	5	3021.2659	-0.005
7	3	5	←	6	3	4	3059.6124	-0.003
7	4	4	←	6	4	3	3077.3871	-0.003
7	4	3	←	6	4	2	3092.0820	-0.000
7	3	4	←	6	3	3	3150.9118	0.002
7	2	5	←	6	2	4	3153.5846	-0.000
8	1	8	←	7	1	7	3249.9108	0.002
8	0	8	←	7	0	7	3251.1110	-0.001
8	2	7	←	7	2	6	3387.6078	-0.003
8	1	7	←	7	1	6	3411.4068	-0.005
8	3	6	←	7	3	5	3485.7387	-0.004
8	5	4	←	7	5	3	3516.4216	0.001
8	5	3	←	7	5	2	3519.4316	-0.000
8	4	5	←	7	4	4	3520.1029	0.000
8	4	4	←	7	4	3	3555.8323	0.001
8	2	6	←	7	2	5	3576.5874	-0.004
8	3	5	←	7	3	4	3619.8626	0.001
9	1	9	←	8	1	8	3647.3454	0.002
9	0	9	←	8	0	8	3647.8308	0.000
9	2	8	←	8	2	7	3789.7264	-0.002
9	1	8	←	8	1	7	3802.2831	-0.003
9	3	7	←	8	3	6	3905.1594	-0.004
9	6	4	←	8	6	3	3952.6284	0.008
9	6	3	←	8	6	2	3953.1281	-0.000
9	4	6	←	8	4	5	3959.7819	-0.002
9	5	5	←	8	5	4	3962.9643	-0.001
9	5	4	←	8	5	3	3972.1068	-0.000
9	2	7	←	8	2	6	3981.2868	-0.006
9	4	5	←	8	4	4	4030.2943	0.001
10	1	10	←	9	1	9	4044.5978	0.009
10	0	10	←	9	0	9	4044.7871	0.007
9	3	6	←	8	3	5	4077.0263	-0.002
10	2	9	←	9	2	8	4189.4072	-0.000
10	1	9	←	9	1	8	4195.4637	-0.002
10	3	8	←	9	3	7	4318.0266	-0.004
10	2	8	←	9	2	7	4372.4087	-0.007
10	8	3	←	9	8	2	4381.4946	-0.022
10	8	2	←	9	8	1	4381.4946	-0.024
10	4	7	←	9	4	6	4394.4162	-0.003
10	6	5	←	9	6	4	4399.2677	-0.000
10	6	4	←	9	6	3	4401.0876	-0.004
10	5	6	←	9	5	5	4409.8153	-0.000

10	5	5	←	9	5	4	4432.8878	0.000
11	1	11	←	10	1	10	4441.7826	0.027
11	0	11	←	10	0	10	4441.8345	0.005
10	4	6	←	9	4	5	4509.6927	0.000
10	3	7	←	9	3	6	4517.2952	-0.006
11	2	10	←	10	2	9	4587.6951	0.005
11	1	10	←	10	1	9	4590.4402	0.000
11	3	9	←	10	3	8	4725.2539	-0.003
11	2	9	←	10	2	8	4758.7222	-0.005
11	4	8	←	10	4	7	4822.5184	-0.007
12	1	12	←	11	1	11	4838.9046	0.015
12	0	12	←	11	0	11	4838.9400	0.023
11	6	6	←	10	6	5	4847.7137	-0.000
11	6	5	←	10	6	4	4853.1851	0.000
11	5	7	←	10	5	6	4855.2144	-0.002
11	5	6	←	10	5	5	4904.5099	0.001
11	3	8	←	10	3	7	4937.5837	-0.009
11	4	7	←	10	4	6	4983.5317	-0.001
12	2	11	←	11	2	10	4985.2675	0.010
12	1	11	←	11	1	10	4986.4586	0.007
12	3	10	←	11	3	9	5128.2036	-0.001
12	2	10	←	11	2	9	5146.5350	-0.004
13	1	13	←	12	1	12	5236.0350	0.026
13	0	13	←	12	0	12	5236.0350	0.016
12	4	9	←	11	4	8	5243.4184	-0.006
12	7	6	←	11	7	5	5282.1745	-0.001
12	7	5	←	11	7	4	5283.2369	-0.009
12	5	8	←	11	5	7	5297.0605	-0.006
12	6	7	←	11	6	6	5297.2967	-0.002
12	6	6	←	11	6	5	5311.4435	-0.001
12	3	9	←	11	3	8	5338.0716	-0.011
13	2	12	←	12	2	11	5382.5085	0.015
13	1	12	←	12	1	11	5383.0081	0.012
12	5	7	←	11	5	6	5386.5078	0.000
12	4	8	←	11	4	7	5442.8430	-0.006
13	3	11	←	12	3	10	5528.2927	0.000
13	2	11	←	12	2	10	5537.5394	0.001
13	4	10	←	12	4	9	5657.3263	-0.003
13	8	6	←	12	8	5	5716.5080	-0.036
13	3	10	←	12	3	9	5724.8417	-0.011
13	7	7	←	12	7	6	5731.8820	0.007
13	5	9	←	12	5	8	5733.3717	-0.009
13	7	6	←	12	7	5	5735.0541	-0.005
13	6	8	←	12	6	7	5746.7570	-0.006
13	6	7	←	12	6	6	5778.7300	0.003
14	2	13	←	13	2	12	5779.6218	0.029
14	1	13	←	13	1	12	5779.8492	0.051
13	5	8	←	12	5	7	5871.9248	-0.000
13	4	9	←	12	4	8	5882.3814	-0.011

14	3	12	←	13	3	11	5926.6933	0.008
14	2	12	←	13	2	11	5931.0893	0.006
14	4	11	←	13	4	10	6065.2099	-0.001
14	3	11	←	13	3	10	6107.3581	-0.007
14	5	10	←	13	5	9	6162.7084	-0.008
14	8	6	←	13	8	5	6165.7140	-0.014
14	7	8	←	13	7	7	6183.2024	0.000
14	7	7	←	13	7	6	6191.5634	-0.011
14	6	9	←	13	6	8	6194.2793	-0.008
14	6	8	←	13	6	7	6257.4103	-0.004
14	4	10	←	13	4	9	6299.5645	-0.014
15	3	13	←	14	3	12	6324.1985	0.015
15	2	13	←	14	2	12	6326.1986	0.011
14	5	9	←	13	5	8	6349.5600	-0.007
15	3	12	←	14	3	11	6492.1598	-0.000
15	5	11	←	14	5	10	6584.4468	-0.008
15	7	9	←	14	7	8	6635.3651	-0.004
15	6	10	←	14	6	9	6637.7628	-0.008
15	7	8	←	14	7	7	6655.0367	-0.001
15	4	11	←	14	4	10	6695.7326	-0.015
15	6	9	←	14	6	8	6746.0734	-0.011
15	5	10	←	14	5	9	6810.1291	-0.016

Table S27: Measured rotational transitions and residuals (in MHz) of (Benzene)$_3$-$H_2{}^{18}O$-H_2O.

J’	Ka’	Kc’	←	J’’	Ka’’	Kc’’	υ_{obs}	υ_{obs}-υ_{cal}
5	1	4	←	4	1	3	2205.3763	0.002
5	2	3	←	4	2	2	2244.6287	0.011
6	1	6	←	5	1	5	2444.7947	0.001
6	0	6	←	5	0	5	2451.3809	-0.002
6	2	5	←	5	2	4	2558.7303	-0.004
6	1	5	←	5	1	4	2613.5283	-0.001
6	2	4	←	5	2	3	2695.9805	0.003
7	1	7	←	6	1	6	2842.4602	-0.005
7	0	7	←	6	0	6	2845.4578	-0.012
7	2	6	←	6	2	5	2968.4479	-0.005
7	1	6	←	6	1	5	3008.5581	-0.001
7	3	5	←	6	3	4	3042.9011	-0.003
7	3	4	←	6	3	3	3128.9146	0.003
8	1	8	←	7	1	7	3239.2298	-0.003
8	0	8	←	7	0	7	3240.5128	-0.007
8	2	7	←	7	2	6	3373.2835	-0.004
8	1	7	←	7	1	6	3397.9353	0.001
8	3	6	←	7	3	5	3467.4783	0.000
8	5	4	←	7	5	3	3495.4573	-0.000
8	5	3	←	7	5	2	3498.1094	0.000
8	4	5	←	7	4	4	3499.4056	-0.002
8	4	4	←	7	4	3	3531.9615	-0.003
8	2	6	←	7	2	5	3557.2974	0.002

8	3	5	←	7	3	4	3595.0320	0.003
9	1	9	←	8	1	8	3635.5713	0.012
9	0	9	←	8	0	8	3636.0854	-0.002
9	2	8	←	8	2	7	3774.4116	-0.007
9	1	8	←	8	1	7	3787.6742	-0.001
9	3	7	←	8	3	6	3885.6723	-0.005
9	4	6	←	8	4	5	3936.9147	0.004
9	5	5	←	8	5	4	3939.1134	0.000
9	5	4	←	8	5	3	3947.1852	-0.005
9	2	7	←	8	2	6	3962.2702	0.002
9	4	5	←	8	4	4	4001.8552	0.003
10	1	10	←	9	1	9	4031.6863	-0.000
10	0	10	←	9	0	9	4031.8906	-0.007
9	3	6	←	8	3	5	4050.4929	0.004
10	2	9	←	9	2	8	4173.0941	-0.002
10	1	9	←	9	1	8	4179.5989	-0.006
10	3	8	←	9	3	7	4297.5612	-0.006
10	2	8	←	9	2	7	4353.4989	0.000
10	4	7	←	9	4	6	4369.7838	0.002
10	5	6	←	9	5	5	4383.2079	0.004
10	5	5	←	9	5	4	4403.7098	-0.004
11	1	11	←	10	1	10	4427.7386	0.007
11	0	11	←	10	0	10	4427.8116	-0.001
10	4	6	←	9	4	5	4477.3527	-0.004
10	3	7	←	9	3	6	4490.2137	0.009
11	2	10	←	10	2	9	4570.3392	0.000
11	1	10	←	10	1	9	4573.3530	0.010
11	3	9	←	10	3	8	4703.9386	-0.000
11	2	9	←	10	2	8	4739.1610	0.002
11	4	8	←	10	4	7	4796.5451	-0.002
11	5	6	←	10	5	5	4870.3797	-0.000
11	5	7	←	10	5	6	4826.1205	-0.007
11	3	8	←	10	3	7	4911.0559	-0.000
11	4	7	←	10	4	6	4948.7558	-0.000
12	2	11	←	11	2	10	4966.8300	-0.000
12	1	11	←	11	1	10	4968.1563	0.000
12	2	10	←	11	2	9	5125.7564	0.004
12	4	9	←	11	4	8	5216.4693	-0.000
12	6	7	←	11	6	6	5264.8156	0.002
12	6	6	←	11	6	5	5277.0808	-0.002
12	3	9	←	11	3	8	5312.6542	-0.005
12	5	7	←	11	5	6	5347.3527	0.003
13	2	12	←	12	2	11	5362.9847	0.018
13	1	12	←	12	1	11	5363.5407	0.008
12	4	8	←	11	4	7	5407.1562	-0.000
13	2	11	←	12	2	10	5515.3679	0.002
13	4	9	←	12	4	8	5847.1842	-0.004
15	4	12	←	14	4	11	6439.4621	-0.006
15	5	11	←	14	5	10	6549.9781	0.002

Table S28: Measured rotational transitions and residuals (in MHz) of $(Benzene)_3$- H_2O - $H_2{}^{18}O$.

J'	Ka'	Kc'	←	J''	Ka''	Kc''	υ_{obs}	υ_{obs}-υ_{cal}
5	1	4	←	4	1	3	2202.0267	-0.006
5	2	3	←	4	2	2	2239.2743	-0.006
6	1	6	←	5	1	5	2447.1758	0.001
6	0	6	←	5	0	5	2453.6966	-0.007
6	2	5	←	5	2	4	2557.3726	-0.000
6	2	4	←	5	2	3	2689.7763	0.008
7	1	7	←	6	1	6	2845.5641	-0.008
7	0	7	←	6	0	6	2848.5630	-0.004
7	2	6	←	6	2	5	2967.5197	-0.001
7	1	6	←	6	1	5	3006.9481	-0.006
7	3	5	←	6	3	4	3039.1634	-0.001
7	3	4	←	6	3	3	3121.4636	-0.002
7	2	5	←	6	2	4	3128.2005	-0.006
8	1	8	←	7	1	7	3243.0779	-0.004
8	0	8	←	7	0	7	3244.3678	-0.004
8	2	7	←	7	2	6	3372.9347	-0.009
8	1	7	←	7	1	6	3397.3505	-0.005
8	3	6	←	7	3	5	3463.6865	-0.023
8	4	5	←	7	4	4	3494.1017	-0.007
8	4	4	←	7	4	3	3524.8918	0.006
8	2	6	←	7	2	5	3551.1841	-0.001
8	3	5	←	7	3	4	3586.1946	0.002
9	1	9	←	8	1	8	3640.1633	0.008
9	0	9	←	8	0	8	3640.6767	-0.011
9	2	8	←	8	2	7	3774.7550	-0.005
9	1	8	←	8	1	7	3787.9848	0.000
9	3	7	←	8	3	6	3882.1390	0.002
9	4	6	←	8	4	5	3931.0873	0.004
9	5	5	←	8	5	4	3932.9111	-0.001
9	5	4	←	8	5	3	3940.4656	0.002
9	2	7	←	8	2	6	3957.2015	0.005
9	4	5	←	8	4	4	3992.7064	0.001
10	1	10	←	9	1	9	4037.0352	0.004
10	0	10	←	9	0	9	4037.2423	-0.002
9	3	6	←	8	3	5	4040.8937	0.012
10	2	9	←	9	2	8	4174.1709	-0.000
10	1	9	←	9	1	8	4180.6973	-0.010
10	3	8	←	9	3	7	4294.4731	-0.004
10	2	8	←	9	2	7	4349.7275	0.000
10	4	7	←	9	4	6	4363.6690	0.001
10	5	6	←	9	5	5	4376.1386	0.008
10	5	5	←	9	5	4	4395.3453	0.001
11	1	11	←	10	1	10	4433.8192	-0.002
11	0	11	←	10	0	10	4433.9010	-0.004
10	4	6	←	9	4	5	4466.2365	0.003
10	3	7	←	9	3	6	4480.5694	0.000
11	2	10	←	10	2	9	4572.1693	0.003

11	3	9	←	10	3	8	4701.4697	0.004
11	2	9	←	10	2	8	4736.5474	-0.001
11	4	8	←	10	4	7	4790.4351	0.006
11	6	5	←	10	6	4	4814.7949	-0.007
11	5	7	←	10	5	6	4818.2963	-0.006
12	1	12	←	11	1	11	4830.5712	-0.005
12	0	12	←	11	0	11	4830.6219	0.012
11	4	7	←	10	4	6	4936.2511	0.011
12	2	11	←	11	2	10	4969.4212	0.008
12	1	11	←	11	1	10	4970.7644	0.004
12	3	10	←	11	3	9	5104.2825	-0.003
12	2	10	←	11	2	9	5124.0754	0.004
12	4	9	←	11	4	8	5210.6285	0.005
13	0	13	←	12	0	12	5227.3412	0.012
13	1	13	←	12	1	12	5227.3023	-0.013
12	6	7	←	11	6	6	5256.0260	-0.004
12	6	6	←	11	6	5	5267.4101	-0.010
12	3	9	←	11	3	8	5305.2148	0.002
13	2	12	←	12	2	11	5366.3124	0.010
13	1	12	←	12	1	11	5366.8901	0.009
13	2	11	←	12	2	10	5514.4882	0.007
13	4	10	←	12	4	9	5624.2970	0.002
13	5	9	←	12	5	8	5692.0520	-0.001
13	3	10	←	12	3	9	5694.1016	-0.006
13	6	8	←	12	6	7	5701.7850	0.001
13	4	9	←	12	4	8	5834.4094	-0.003
14	3	12	←	13	3	11	5902.4390	0.005
14	2	12	←	13	2	11	5907.4275	0.002
15	4	12	←	14	4	11	6435.5610	-0.003
15	6	10	←	14	6	9	6587.2008	-0.007

Table S29: B3LYP-D3BJ-cc-pVTZ equilibrium coordinates (Å) in the principal axis system of Bz_2-(H_2O)-I (minimum)

27

Coordinates from ORCA-job 312-reactant PAS

C -0.8676064657844664 1.0616655974048226 -0.11408459201919095

C -1.4290131397779162 0.8217821706154314 1.135743886370888

C -2.7326336992257545 0.34850115443783164 1.235596576320195

C -3.4764854097062554 0.1120145402926453 0.08344780748426299

C -2.915298005743257 0.3499125745202201 -1.1670232990718037

C -1.612407171639719 0.8262174873637524 -1.2650223838646786

H 0.15011303599003126 1.4170610456754762 -0.1864189954716284

H -0.8434249188227141 0.9922974843875695 2.028455350949867

H	-3.1671039376075893	0.16051427946752111	2.208190230842942
H	-4.490905468412019	-0.25613245553096664	0.16015335591898236
H	-3.491079181927394	0.16002962465382853	-2.0626157090771837
H	-1.1726517796166513	1.0045373005112144	-2.2368358535639974
O	-1.000193959485991	-2.4547950634414613	-0.01112518281968356
H	-0.6446499279533732	-1.5676923653971395	-0.13420087264871294
H	-1.9216892424538439	-2.292576435324125	0.2119491816970793
C	2.343012727095393	0.08304045617775017	1.400105032620104
C	1.879452346175394	-1.0702883064093633	0.7770496425564922
C	1.9488573131241493	-1.1873835396031494	-0.6072221184253153
C	2.478240431504452	-0.1499168737150831	-1.3672511142607613
C	2.9404013415567505	1.0041724502192535	-0.7441625332303716
C	2.8734395688989194	1.1203271124635619	0.6406732485620551
H	2.289608337359825	0.17548480012916676	2.476740130637731
H	1.446388984165526	-1.8712587361861603	1.359189820406496
H	1.5783423974069342	-2.0823437581932724	-1.0877343840279705
H	2.5296523860773776	-0.24101182613381292	-2.443965442396483
H	3.352481703751628	1.810956201113198	-1.3354806150910568
H	3.2324348003751853	2.0183090123206053	1.125668098927329

Table S30: B3LYP-D3BJ-cc-pVTZ equilibrium coordinates (Å) in the principal axis system of Bz_2-(H_2O)-I (Transition State)

27

Coordinates from ORCA-job NEB-Final PAS

C	-0.8665696500648964	1.0652829256228564	0.013054801625841876
C	-1.5280067245216222	0.8244256565970917	1.2128922510070383
C	-2.833588140465107	0.3451298036901134	1.2026911086203211
C	-3.4746313636436654	0.10834521635058372	-0.009173727245576786
C	-2.8134086303113626	0.3497580876170006	-1.2091607499290353
C	-1.5085540466708727	0.8281563702844389	-1.1968384201838493
H	0.1540590080379389	1.4231178852127835	0.019832196699209093
H	-1.0208041768912817	0.9978002016808533	2.1531815186495207
H	-3.3458243156449505	0.15285612862658138	2.1356598995966145
H	-4.492469991141194	-0.2612238674792411	-0.017529930027884475

H	-3.3117942495426376	0.16298007554468538	-2.1508375005714946
H	-0.9880974987645226	1.0051350635543033	-2.1272560985289197
O	-0.994612239504792	-2.4465060473711695	-0.0024153332045522002
H	-0.6319792118204379	-1.5536268841030112	0.012630433117897983
H	-1.9460187435299847	-2.306445704440846	-0.023858873059032493
C	2.4158334257352685	-0.04707114125308751	1.3914980802301522
C	1.9150280173562761	-1.1372354392923831	0.6872111508488186
C	1.908147340106074	-1.1250403802095605	-0.7038214569164051
C	2.401290182397511	-0.022920675405749356	-1.3940263683443008
C	2.9002854911649263	1.0662116982649574	-0.6902042331506891
C	2.907845442842498	1.0547923318539265	0.6998758092943486
H	2.421072045756492	-0.05616939912249347	2.4729506883650245
H	1.5148491150593668	-1.9903890565713473	1.2159182812957825
H	1.5028105040510311	-1.9683748174777493	-1.2440906610536382
H	2.394741011242124	-0.01366889890404127	-2.4754730486365313
H	3.28503941570783	1.9232593737009318	-1.2273891413058022
H	3.2982243326142595	1.9029444931159247	1.2469888978233756

Table S31: B3LYP-D3BJ-cc-pVTZ equilibrium coordinates (Å) in the principal axis system of Bz_2-$(H_2O)_2$-I

30

Coordinates from ORCA-job 5 PAS

C	-2.589021763764773	-0.3111066462453446	-1.0182910310956579
C	-1.309056309365756	0.18313065326090933	-1.2402513630758145
C	-0.860444639985484	1.297903294985985	-0.5403692928545811
C	-1.6928341090757528	1.9194906808968284	0.38535908689995596
C	-2.9745654217488973	1.426440876212283	0.6100269972489041
C	-3.422900098629605	0.310502314130805	-0.09317213092622259
H	-2.920164547706243	-1.1965437337020286	-1.5419107133198617
H	-0.6524696427827161	-0.3195500416600582	-1.9346237272566247
H	0.1416751415957191	1.6668241335243628	-0.7053845407746188
H	-1.3412573860941277	2.7826111924368706	0.9344809228629531
H	-3.621514232470582	1.9071260526055938	1.331659368968511
H	-4.418948881597988	-0.07411724984335681	0.08202916777127865

O	-0.5459656253455196	-3.0259970510112426	-0.41375027100184164
H	-0.6277256899485021	-3.933265785590239	-0.10877653618765243
H	-0.7207564646010393	-2.481319854058872	0.375025465903115
C	2.4767461612088018	0.0959298651691087	-1.3980397397927096
C	2.0365013602263713	-0.9822547967798546	-0.6400032609026143
C	2.0685864826598777	-0.9081330522760841	0.7492104031432315
C	2.5205563996150717	0.24683488905777334	1.3791968432637638
C	2.951337281721809	1.329377334156743	0.6179956609391638
C	2.935393201423749	1.2505850259260733	-0.7700576378227044
H	2.456025253074458	0.04135646170412068	-2.478421079590509
H	1.6422915203810853	-1.87137572880216	-1.1105106402366651
H	1.737683620151235	-1.7557086391358292	1.3337110686017302
H	2.5430050019732464	0.3016434486075173	2.459608623563485
H	3.303723470072974	2.2287006045639908	1.1051577882193808
H	3.2765305929969117	2.089208336819625	-1.362568180712281
O	-0.9782877941693753	-1.241490786131792	1.7917667610301495
H	-0.2919512528106939	-0.6173535235605663	1.5256198495010587
H	-1.7993066726096996	-0.7986491926015624	1.5430607253651465

Table S32: B3LYP-D3BJ-cc-pVTZ equilibrium coordinates (Å) in the principal axis system of Bz_2-$(H_2O)_2$-II

30

Coordinates from ORCA-job 44 PAS

C	-2.87107693300084	1.5952013940053238	0.4554395140263927
C	-2.4613761231928724	0.6325226819641282	1.3712056461801485
C	-2.0188450721574287	-0.6081261375058636	0.9233102631694396
C	-1.9793342634592783	-0.8894131937817403	-0.4390057952067121
C	-2.3948923260338835	0.07408750814203215	-1.3518305772346473
C	-2.841071703395527	1.313965507173807	-0.9069146286640379
H	-3.215313964551769	2.5607282419212094	0.8011965793580932
H	-2.487132412160616	0.8484648645771908	2.43099836000177
H	-1.7047534145727168	-1.360168981359776	1.6349595282072624
H	-1.6067467547757204	-1.848437417885474	-0.7734362440213512

H	-2.3702176709614475	-0.14305349076372645	-2.4115411943612086
H	-3.162853787073632	2.061781090612809	-1.6194724208627773
O	-0.06471930176904107	-3.621192702713263	-0.3217545115399217
H	-0.5152156893731955	-4.263911483288589	0.23238233983697448
H	0.3629146976772599	-3.007825434451744	0.30273950404174804
C	3.0499777652424926	1.1854224686081158	0.7868873261523637
C	1.7587768098202192	1.7027351678481575	0.7545539257643257
C	0.9000433298934588	1.3669450357542927	-0.2869761419599003
C	1.3337949315675723	0.5140401986193258	-1.2967062167258647
C	2.623473038210502	-0.003897947034463674	-1.2666826905535864
C	3.482947065135616	0.33242746914471966	-0.22413079059960228
H	3.7167248921476674	1.4444999434362111	1.5982425047558853
H	1.4189860129386411	2.359740687494502	1.5435986925476297
H	-0.10911388445715492	1.7518797013399874	-0.3092956745133852
H	0.6556026612058407	0.23780838417422628	-2.0911904599557385
H	2.9554849407183017	-0.6741362095397804	-2.047545948986778
H	4.487438800564524	-0.06882878858777174	-0.20158116723076214
O	1.0701887156383079	-1.6425134727011144	1.3530762554004014
H	0.5325395928651534	-0.8877145667253323	1.0828658920295775
H	1.9630503631376104	-1.3905956292467962	1.0914844238732404

Table S33: B3LYP-D3BJ-cc-pVTZ equilibrium coordinates (Å) in the principal axis system of Bz_2-$(H_2O)_2$-III

30

Coordinates from ORCA-job 12 PAS

C	-3.2031715441405444	1.0109553244780718	-0.7409006254824215
C	-2.0374926482627225	1.7679537123124054	-0.6507234805797176
C	-1.0973149159410596	1.481130132339562	0.3347318024818052
C	-1.3226998284392248	0.43825018119230097	1.2267713928530537
C	-2.48306153085283	-0.32159288610624087	1.135695247682497
C	-3.425063378826733	-0.033708978454971505	0.15342052290766786
H	-3.934227121685713	1.2305545152897528	-1.5075887554825682
H	-1.8594859718024683	2.571383396823918	-1.3530197335643288
H	-0.1806291271795762	2.050930616128411	0.39663141524443035

H	-0.5830169210608311	0.20296013735052226	1.9771813951528534
H	-2.6366734146689477	-1.151787687769068	1.8101134565468102
H	-4.327809241688054	-0.6250996587579541	0.08018251817222986
O	-0.6816411304144524	-1.1812170006533562	-1.7219347935784868
H	-1.4484554501055915	-0.6514856797844235	-1.4715566489060736
H	0.07404185776600473	-0.6393028354790624	-1.4635899766817224
C	2.715524058906084	1.4833438787414792	0.6702315528563956
C	2.2766156305058955	0.40657644278581245	1.434945842318302
C	2.0487785504571483	-0.8273390062478294	0.8372014682867809
C	2.2770571742713215	-0.9878946291232057	-0.5261062053472088
C	2.71645011737726	0.08738108381985474	-1.2927644405618024
C	2.9309824782962544	1.3260167421146	-0.6945277890349005
H	2.8884271800073167	2.4439232412713885	1.1374256720842406
H	2.103816387843179	0.5331193133093702	2.49563220882591
H	1.6672719037822175	-1.6618670289695274	1.4072755492222697
H	2.0990080799435566	-1.9521326657426643	-0.9817354320770763
H	2.8949117588038202	-0.041121254811783185	-2.3521630533139772
H	3.2704492113532706	2.1632389527429625	-1.2896709513172695
O	-0.29971748854570446	-3.070465713467817	0.3917907569192344
H	-0.5094493737870082	-2.4814928316730294	-0.3553314850295312
H	-0.5724328071149328	-3.944105711637785	0.10076666052936387

Table S34: B3LYP-D3BJ-cc-pVTZ equilibrium coordinates (Å) in the principal axis system of Bz_3-(H_2O)-I

39

Coordinates from ORCA-job 22

C	-2.7612085463632914	-2.7328698076694242	-0.2580269422763942
C	-1.6869557749879311	-2.3219184207270573	-1.039262846727863
C	-1.4561962072069363	-0.9667560829258579	-1.246618005222396
C	-2.30079561945376	-0.02035840273093841	-0.6774866962764357
C	-3.378752916956961	-0.4308681671567095	0.09980557067136907
C	-3.609154933319894	-1.7869205153217123	0.3111950934570899
H	-2.938235368524815	-3.787015059243547	-0.09238741867972362
H	-1.026041394100599	-3.057558965059483	-1.4778250916999973

H	-0.6094578296079476	-0.6425160793382741	-1.8344304236719216
H	-2.106468291632558	1.0311857854806226	-0.8264184755045331
H	-4.031744153150284	0.30586906765865207	0.5478328207871114
H	-4.447136492629588	-2.106683823754277	0.9162368810243445
C	4.092884300979633	-0.39548238095265414	-0.29076523095474666
C	3.403139304391186	-1.0660984177012678	-1.2953619539740524
C	2.223376471770017	-1.7399038211597422	-0.9962497561133612
C	1.732006778067329	-1.7426398752169279	0.30403058323357574
C	2.422772571785229	-1.0739347279665354	1.308602973523477
C	3.6029718902705206	-0.4014034990794948	1.0115822174090292
H	5.0086258225479225	0.13181641874260172	-0.522510800183749
H	3.7830985272811573	-1.061236569056325	-2.3083498012521475
H	1.6862967912319902	-2.26056577906148	-1.7782719334154626
H	0.803474273941339	-2.239301785266158	0.5423262292679248
H	2.0238187320089938	-1.0741643834882943	2.313341378083322
H	4.138627922973131	0.12282432888402235	1.7917949537230327
O	-0.8065885669118397	-0.9459176558611533	2.2964178631802876
H	-1.6484788409857793	-1.375091131393211	2.111605588589001
H	-0.7003218880699427	-0.3434146711801861	1.5517037426310016
C	-0.7564622744794092	3.3912921781693046	-1.094179160170407
C	0.3183141165488695	2.5449266568948077	-1.3474918689767077
C	0.8920397578025475	1.8168266740110384	-0.31101791530136375
C	0.3897376423438011	1.937976899286091	0.980226326990429
C	-0.6906934108676459	2.7765239268800164	1.2337551188916733
C	-1.2634708906915193	3.504927578812308	0.1964300987745238
H	-1.1996453725842733	3.9596796015041105	-1.9010384353453689
H	0.7096769137897818	2.45387070898622	-2.352004662536016
H	1.7254974935448184	1.157809333676866	-0.5044725085585884
H	0.842774430051304	1.3789978197065822	1.7869527771953158
H	-1.083956643185123	2.85938591115207	2.237851889312716
H	-2.102544433814801	4.159171688574151	0.3923936503464752

Table S35: B3LYP-D3BJ-cc-pVTZ equilibrium coordinates (Å) in the principal axis system of Bz_3-(H_2O)-II

39

Coordinates from ORCA-job 18 PAS

C	-3.4473388012294537	-0.5273514370610873	0.24385714003122083
C	-2.453645531714269	0.0309188688818204	-0.5546616741700469
C	-1.5681011329754742	-0.7944770195204305	-1.2371171421247544
C	-1.6758978971046494	-2.1761317149232537	-1.12271284196661
C	-2.6621822936262167	-2.734913524224107	-0.3179904921712419
C	-3.5508103998151475	-1.9097974465857372	0.3655157022939451
H	-4.133117583537664	0.11527528071659596	0.7793070801495134
H	-2.360964711893259	1.1043006407629443	-0.6283381615675289
H	-0.7869588586380698	-0.35782508915813416	-1.8422680735476167
H	-0.9850262942749503	-2.818218016377766	-1.6521732547957566
H	-2.739321108911481	-3.8094340720335684	-0.22173223034018547
H	-4.31998087674931	-2.3427612191836453	0.990891953645455
C	4.066486935186572	-0.5297394173720853	-0.29561092113689114
C	3.3357019819031497	-1.160952028988887	-1.2967623989874446
C	2.1215342857329094	-1.7680556848981739	-0.9923058154906538
C	1.6378188062726653	-1.7449589244547183	0.31046568638644817
C	2.3709777762287416	-1.118323895631499	1.3120626056277929
C	3.5847580255901352	-0.5112453499248133	1.0097735845816058
H	5.00855723105869	-0.05351823202247872	-0.5321939999088381
H	3.710562889859651	-1.1768934353139695	-2.311544098931454
H	1.5512637785914736	-2.2551556221580076	-1.772105463833277
H	0.6835993640696914	-2.187395395317869	0.5530737454138296
H	1.9789791409458652	-1.0996993569908393	2.319408469344639
H	4.1528920571175565	-0.019381286924622022	1.7881187459338466
O	-0.8116210030458729	-0.7840755337328523	2.363794040090137
H	-1.6012044861405212	-1.1481859341395868	1.9486527116831918
H	-0.5468864137630928	-0.07995758774644969	1.7618204091511898
C	0.31897287259994367	2.4410591660638796	-1.4098582324520423
C	0.9689639399603283	1.8091709548024792	-0.35596006228931465
C	0.5503930219513052	2.038194788082831	0.950582129952021

C	-0.5195137521402482	2.890269872432112	1.2037303461718416
C	-1.1673624849733493	3.523114777452416	0.14802076902126823
C	-0.7469919169567507	3.2990106126823484	-1.158781265559134
H	0.6440674581425418	2.265906875038833	-2.42677976454239
H	1.7949377481736173	1.140334228483298	-0.5469989705836465
H	1.067510273080217	1.5567827214869687	1.7694303208466617
H	-0.8460286535165149	3.0592577365132105	2.220879079709117
H	-1.997718343492267	4.188549092690382	0.342761218125359
H	-1.2508801392872704	3.790724822367642	-1.980240803807108

Table S36: B3LYP-D3BJ-cc-pVTZ equilibrium coordinates (Å) in the principal axis system of Bz_3-$(H_2O)_2$-I

42

Coordinates from ORCA-job 1 PAS

C	3.1438153837567806	1.114304670046524	1.847146892370454
C	3.917019255661082	0.20402175979969825	1.1337885540002612
C	3.7444824298123525	0.07435412613189603	-0.24066563349045064
C	2.799490036029924	0.8519028005858392	-0.9004943229832434
C	2.025792737862823	1.7612004517899942	-0.18784240481739056
C	2.1988561368872745	1.8921600503434257	1.18530703006443
H	3.2764706244668287	1.2153977784233159	2.916104089713914
H	4.651134758115117	-0.40185133128568995	1.6480246794900302
H	4.348129299927033	-0.6313504195875418	-0.7963236567843102
H	2.6526569641073774	0.7642559872797027	-1.9674927874496209
H	1.296218938280241	2.3535912779740094	-0.719830516136279
H	1.5968603559249834	2.597950683820187	1.74232580904339
C	-0.7445020473551381	-1.4779562087777793	1.0447294871277828
C	-1.5661541575669649	-2.4737016108380137	0.5259380107970004
C	-1.0141888203570382	-3.536389440388192	-0.18084391749491316
C	0.3625317340710942	-3.6030929170711703	-0.3697493919483335
C	1.1849104400332706	-2.6074519498255375	0.15032277009095216
C	0.6317383254678537	-1.544320000059982	0.8579752796652244
H	-1.1794221358065342	-0.6445655283950438	1.5766433065246426
H	-2.6365597274593213	-2.4118795452280724	0.6654980238062081

H	-1.6542679502803164	-4.306179515243941	-0.5901084895428973
H	0.7930363931621816	-4.427714212674051	-0.9220538498863851
H	2.255435895885818	-2.6533230742616154	0.00451714980254093
H	1.2697839342809158	-0.7656956286609106	1.2471482308478932
O	0.43101687718196907	1.8484405721744568	-3.0223096149888673
H	0.18492044921672612	0.9186452090430635	-2.8528410882887902
H	0.36518701416919697	1.9626521667793948	-3.973436133805559
C	-3.0733439934731384	0.8319548318084847	-0.864478165822773
C	-2.022788927261872	1.724831562030013	-0.6753768859042798
C	-1.747850142765478	2.2038389357098063	0.6015740816283184
C	-2.522336415180964	1.7983602454311707	1.682103348896114
C	-3.5723219665709016	0.9049614902274277	1.4902280422147343
C	-3.8470105605780613	0.42072283677906175	0.2167650267814421
H	-3.292554014379389	0.460160482147265	-1.85707144594575
H	-1.4166645015336008	2.0383457498307083	-1.5145804870448454
H	-0.9292815341392858	2.8940590154335495	0.7509509348146156
H	-2.3077298415613727	2.1745882786816666	2.6735577214198583
H	-4.173732981371638	0.5875203884877448	2.331557305192021
H	-4.6640513866625595	-0.2719005788322106	0.06476063492369419
O	-0.3413179335180016	-0.7284996347757575	-2.2779802282318324
H	0.3348054911751065	-1.2684545841946655	-1.8510372453008501
H	-0.9811272385126221	-0.5710055436451275	-1.5723183309819648

Table S37: B3LYP-D3BJ-cc-pVTZ equilibrium coordinates (Å) in the principal axis system of Bz_3-$(H_2O)_2$-II

42

Coordinates from ORCA-job 1 PAS

C	2.913252577103006	-1.573441649560331	0.2954039825455016
C	3.273357588584359	-1.1693840095037658	-0.9868562197652611
C	4.224860043648503	-0.17013779191582457	-1.1660162116864172
C	4.822812703453729	0.42529040250824485	-0.06103485592134542
C	4.466554790374975	0.02169385955123199	1.221873988201989
C	3.513112349993993	-0.9747630942465382	1.3982294831852529
H	2.1561722305807622	-2.3346199003543586	0.4272594034956749

H	2.812408428178549	-1.63649254973914	-1.847321371698348
H	4.500012165454264	0.14190593460440895	-2.164685156758197
H	5.565580403429736	1.1998553186705312	-0.1984534540538047
H	4.931644320059967	0.4847701806188232	2.0820935620522802
H	3.2345418333688873	-1.2863075621619224	2.3962042612391095
C	-2.918666579030766	-1.4522136023083676	0.5108341561069857
C	-3.6461498937211663	-0.7587514738172048	1.4720430762647754
C	-4.618161642121638	0.1587216089230209	1.0866148380668301
C	-4.866430910859634	0.38358495033605217	-0.2637942038727889
C	-4.141910599880365	-0.3106116969193204	-1.2269918032067209
C	-3.170794216924525	-1.227398192255354	-0.8386986912706587
H	-2.1481506989490513	-2.1562121499636966	0.793603308917041
H	-3.4547390779524254	-0.9329867625181362	2.5226817023294474
H	-5.181555983506368	0.6983741923664308	1.8362664011376897
H	-5.622364372720168	1.096994835024923	-0.5639176481337356
H	-4.334559824310553	-0.13747837373922298	-2.277466082558514
H	-2.6047486933050417	-1.7692793835147531	-1.5840715943827681
O	-0.07208709582219322	-3.1382301618521655	-0.11360021333236053
H	-0.026117376571947633	-2.315278585659479	-0.6383263415801275
H	-0.15418205810753005	-3.8442338271330683	-0.7599115653586856
C	1.4119633341942124	2.0883074421148393	0.09270212863402956
C	0.6344161086077457	2.658766852591811	-0.9099153039038227
C	-0.7524989673626116	2.5525659981355115	-0.8607085163487649
C	-1.3632665428994237	1.8757445909343682	0.19087312694267747
C	-0.583856144383329	1.310995802732888	1.196359548423121
C	0.8009588073164896	1.4177631391028265	1.148227913613003
H	2.4897494766251795	2.1551968194851088	0.05084038747601322
H	1.1083988968547136	3.1781473456740463	-1.7319231774691655
H	-1.3573226367476598	2.990397322116222	-1.6434988990035568
H	-2.4389084972853072	1.7788721032000472	0.22517156903486554
H	-1.060762863272603	0.776638696953386	2.0054492920323987
H	1.4093749496372912	0.9699624400382353	1.9202620015735734
O	0.06861045852666896	-0.7191779152789066	-1.5132834632946557
H	-0.6395256253605385	-0.14375884747668805	-1.1973587731569664

H	0.8755564217204016	-0.28562261647982157	-1.2086159683441786

Table S38: B3LYP-D3BJ-cc-pVTZ equilibrium coordinates (Å) in the principal axis system of Bz_3-$(H_2O)_2$-III

42

Coordinates from ORCA-job 11 PAS

C	2.8958915933906106	2.1514402761267712	0.9160639927547611
C	2.94375720823981	0.7641738659933938	0.8176541068044443
C	2.633162776676986	0.13684321129149205	-0.3850428255573852
C	2.2730223523456514	0.9018938167931657	-1.4896738884857224
C	2.2285923767115885	2.2879676194720004	-1.3942565511615566
C	2.5389006373679246	2.913679470248657	-0.19095429808456393
H	3.1360617952086156	2.637386991885096	1.8522022176572006
H	3.225304779057325	0.16263327458078855	1.6710462776139519
H	2.6748925478851913	-0.9413706193289731	-0.4406898627373221
H	2.0318906837523034	0.41659710273767186	-2.4259331261932693
H	1.9545115893612688	2.8818122748456023	-2.256143471287303
H	2.4997100733204918	3.9919619690865424	-0.11593879463105787
C	-0.015163039080837269	-3.453320243771446	-0.7223791163130213
C	0.20209669513938153	-2.3306898967105467	-1.5129640363229586
C	-0.735990724882402	-1.3053268880904718	-1.5433706714230997
C	-1.8976977221040026	-1.4004271137043856	-0.7854365206986081
C	-2.119553654009776	-2.525979857937407	0.002362033040704454
C	-1.1781164417722625	-3.552391008711286	0.033507411541171636
H	0.7349526931030479	-4.228622233129922	-0.669466699667068
H	1.1129257083207287	-2.2510793347407514	-2.090235183769777
H	-0.5584657531661633	-0.4210095839863997	-2.138984693113273
H	-2.616074025678443	-0.5942726613000234	-0.7959968458898715
H	-3.021787595816289	-2.6002165415427343	0.5948179073365533
H	-1.3454819397537023	-4.421828135272795	0.6549306798310977
O	0.432986611786072 56	-1.1852701604533962	1.8662349041651045
H	-0.35071030332226505	-1.548353398980625	1.432890200492211
H	0.6977001244566443	-0.45026902662147894	1.3001432514058302
C	-0.9501700028154847	1.8314606558131854	0.5281685699881331
C	-1.4962519549067665	2.2617248428645835	-0.6755034742986927

C -2.874337140751941 2.247798762402711 -0.8596309284083818

C -3.7068355017311223 1.8047936224750443 0.16349607671414118

C -3.159709622779408 1.3725011834673824 1.3666780773759786

C -1.781082183273547 1.3844780677797799 1.5490002336006476

H 0.1217527338976799 1.8512544713147414 0.6636446277934789

H -0.8432729339825495 2.6061788156698915 -1.4655545894190634

H -3.2997545391209946 2.5817263950050484 -1.7965994158071723

H -4.779388853453221 1.7956968410373868 0.022119972226118313

H -3.807130827967487 1.0252340817710472 2.160781747635966

H -1.3531662977836105 1.0329784551178944 2.4774650937668623

O 2.6396918763615838 -2.818072333300844 1.143301134781288

H 3.1713766076659753 -3.0113340961818373 1.919192869885148

H 1.8510569652071351 -2.355030722784202 1.4804998672249596

Table S39: B3LYP-D3BJ-cc-pVTZ equilibrium coordinates (Å) in the principal axis system of Bz_3-$(H_2O)_2$-IV

42

Coordinates from ORCA-job 22 PAS

C 3.6626919495968706 0.5331993286674618 0.49427655800536663

C 2.440281364929107 0.1422657536572714 1.0312203610265016

C 1.398214254062784 1.057888186633907 1.127940800668531

C 1.5794759834099712 2.3644212874063615 0.68605326428804688

C 2.802065727564863 2.7555910569408484 0.14723671086534207

C 3.844566312008521 1.8382649073481867 0.05114811459594675

H 4.468556614625425 -0.18323322913158038 0.4102734498455841

H 2.290572763854544 -0.8786915303372427 1.3503632149825509

H 0.44107926024644656 0.7445802681135224 1.5172078259139279

H 0.7697104356134468 3.077686559397502 0.7552575161342119

H 2.9419913029070694 3.772083242839175 -0.19554905461476813

H 4.7928377727855755 2.140360772026369 -0.37225225135325773

C -0.12570045992521986 -2.468336585385913 1.668665336700758

C -0.8088047511089493 -1.7833640305915848 0.6707635301397137

C -0.40150341477252544 -1.8973603407415522 -0.655135635693748

C 0.6955243145076455 -2.6911887633346003 -0.9762559171896514

C 1.3815511228421533 -3.375513405155937 0.023216619437798295

C	0.9687568616645066	-3.2656983260239523	1.3460103530291694
H	-0.4449972855957695	-2.3816568805834493	2.6988253201100663
H	-1.6580175525586822	-1.1634328252863748	0.9194878259997873
H	-0.9314952973253291	-1.3735360339096154	-1.4380126938309987
H	1.012003441701799	-2.7796639065987634	-2.0073414940696197
H	2.231071301880445	-3.9954345685744137	-0.23075515358819182
H	1.4976713354031344	-3.798923897161103	2.124980550795954
O	1.4059425126215326	0.4782792224253347	-2.209694393625293
H	1.6836131286945613	1.2891036547123544	-1.768514272991627
H	1.339098454896126	-0.16569900645195448	-1.4935780201032949
C	-1.9867777506777833	1.6265186152028583	-0.34168855480408405
C	-2.8637248398227926	0.7525009559634267	-0.9739906349697438
C	-3.8931899275429185	0.16342447884806915	-0.24764915694259348
C	-4.045190896699808	0.44603455798398467	1.106056057116153
C	-3.168823265322355	1.3237156560136385	1.7356523337048397
C	-2.140803964443152	1.9153944663711437	1.009268090689795
H	-1.1760804454852423	2.066633895754806	-0.9057287551749064
H	-2.7191569834481633	0.5216389121632649	-2.019733521814199
H	-4.572651544544038	-0.5227868323820225	-0.7352278245096509
H	-4.843190938856142	-0.018384241995273873	1.6700667707848666
H	-3.2855327107799037	1.5444452630844006	2.7883052957467798
H	-1.4638023891265624	2.6014737424176646	1.5008023290066956
O	-0.9986745409864989	-0.0798362821302815	-3.527904829078183
H	-0.14053944061046225	0.21268931339510955	-3.1706326578739765
H	-0.8213056450680747	-0.3383097303054628	-4.435526516702889
